# Sexual and Gender Minority Migrants' Experiences of Health Service Access and Utilisation: A Qualitative Meta‐Synthesis

**DOI:** 10.1111/jocn.17683

**Published:** 2025-02-14

**Authors:** Maria Gottvall, Osszián Péter‐Szabó, Rummage Isaac, Christoffer Aav, Erik Norgren, Tommy Carlsson

**Affiliations:** ^1^ The Department of Women's and Children's Health, CIRCLE – Complex Intervention Research in Health and Care Uppsala University Uppsala Sweden; ^2^ The Department of Health Sciences The Swedish Red Cross University Huddinge Sweden

**Keywords:** asylum seekers, forced migrants, health communication, LGBTQ+, nurse–patient relations, sexual and gender minorities

## Abstract

**Aims:**

To describe and synthesise qualitative studies exploring sexual and gender minority migrants' experiences of health service access and utilisation.

**Design:**

Systematic meta‐synthesis of qualitative studies.

**Data Sources:**

Systematic searches in four databases and citation screening were conducted in 2023 and 2024. English‐language empirical qualitative studies published in scientific journals within 10 years were included. Of 1109 screened, 21 reports were included.

**Methods:**

Included reports were appraised using CASP and JBI checklists. Extracted results were analysed with inductive content analysis in a collaborative process.

**Results:**

All reports had acceptable quality, including 365 participants from 72 countries. A range of external and internal barriers to accessing health services were reported, including financial constraints and fears. Although migrants expressed an appreciation of health services, they also experienced non‐affirming behaviours and discrimination related to their intersecting identities. Several essential components in health services necessary to cater to the needs of migrants were addressed, including the personality and manner of health professionals as well as adherence to confidentiality.

**Conclusion:**

Barriers to health services, intersectional discrimination and non‐affirming behaviours when interacting with health services are pressing issues that need further attention.

**Implication for the Profession and/or Patient Care:**

Ensuring safety through affirming support is key to achieving high‐quality and accessible health services for sexual and gender minority migrants. Nurses and other health professionals need to carefully consider intersectional layers related to sensitivity and safety when supporting sexual and gender minority migrants. Open, friendly, validating, respectful and encouraging communication is essential in clinical settings.

**Impact:**

This review addressed experiences of health services amongst a marginalised population. The findings highlight the importance of affirming care and are relevant for health professionals, stakeholders and decision‐makers.

**Reporting Method:**

ENTREQ.

**Patient or Public Contribution:**

Two persons with lived experience were involved in the meta‐synthesis.

**Summary**

- What does this paper contribute to the wider global clinical community?
  ○ External and internal barriers impair access to health services, calling attention to the need for further efforts to improve accessibility for marginalised populations experiencing minority stress.
  ○ Sexual and gender minority migrants experience discrimination and non‐affirming behaviours when interacting with health services, calling attention to the need for further improvements in clinical support.
  ○ It is essential to ensure an open‐minded, friendly, validating, respectful and encouraging approach that adheres to safety and confidentiality when supporting sexual and gender minority migrants.

## 1 Introduction

Globally, the numbers of forced migrants are increasing and have reached an all‐time high prevalence (UNHCR 2023). Whilst people may seek asylum due to a range of circumstances, many forcibly displaced persons do so because they face significant dangers in their country of origin. Despite calls for global action by international organisations (ILO et al. 2015), sexual and gender minorities (SGM) face considerable violence, threats and discrimination that begins in childhood and continues throughout their lives (Balsam et al. 2005; Blondeel et al. 2018). The victimisation and violence against SGM individuals is so widespread and severe that affected persons need to escape and seek protection in other countries (Alessi et al. 2021). Many countries acknowledge well‐founded fear of persecution based on sexual orientation and gender identity as a valid reason to be granted asylum (UNHCR 2012).

Needing to flee to another country and seek asylum is a significant life event impacting the health and well‐being of millions. In comparison to non‐migrant populations, forced migrants have a higher incidence of mental health disorders (Kirmayer et al. 2011; Lindert et al. 2009) and chronic diseases (Kumar et al. 2021). Forced migrants experience high rates of post‐traumatic distress, depression and anxiety linked to traumatic events experienced before and during migration (Nissen et al. 2021). Despite various health‐related burdens whilst resettling in a host country, the wider population of forced migrants experiences unmet needs of support (Lebano et al. 2020). When able to access health services, they are at risk of encountering language barriers and discrimination, calling attention to the need to ensure culturally appropriate support (Mangrio and Sjögren Forss 2017).

In the last decade, reviews have consistently shown higher rates of mental health burdens, including depression, anxiety, substance dependence, self‐harm and suicide ideation, amongst SGM individuals (Dhejne et al. 2016; King et al. 2008; Plöderl and Tremblay 2015). The minority stress model presents a possible explanation for the burdens experienced when having a non‐heterosexual and/or non‐cisgender identity, highlighting the consequences of being subjected to societal oppression and stigma (Meyer 2003; Pitoňák 2017). Studies confirm that exposure to discrimination, stigma and violence can explain the higher levels of mental health burdens amongst SGM populations (Bränström 2017). However, SGM individuals experience doubts about the level of cultural competence and friendliness of their health providers (Gahagan and Subirana‐Malaret 2018). Studies show that registered nurses and student nurses feel uncertain about how to adequately support SGM individuals (Gahagan and Subirana‐Malaret 2018; Gottvall, Brunell, Eldebo, Kissiti, et al. 2023). There is a need to increase cultural sensitivity in supporting SGM within health services (Baptiste‐Roberts et al. 2017).

## 2 The Review

SGM‐forced migrants experience a range of societal disadvantages when resettling in the host country (Gottvall, Brunell, Eldebo, Johansson Metso, et al. 2023). Through an intersectional perspective, belonging to more than one oppressed or marginalised population can result in unique stressors impacting health and wellbeing (Lee and Brotman 2013). Literature reviews highlight the impactful health‐related burdens experienced in the post‐migration period, including the risk of developing persistent and serious disorders (Gottvall, Brunell, Eldebo, Johansson Metso, et al. 2023; Yarwood et al. 2022). Nurses and other health professionals are responsible for ensuring that all patients are treated in a respectful and non‐discriminatory manner (International Council of Nurses 2021). Several organisations call attention to the importance of competence development amongst health professionals, regarding the health needs of SGM individuals (Sherman et al. 2023). Many studies investigating the health and well‐being of SGM migrants use qualitative methods, providing valuable in‐depth and context‐based information drawn from lived experiences. However, limited efforts have been made to synthesise the qualitative research exploring how encounters with health services are experienced from the perspectives of SGM migrants.

## 3 Aim

To describe and synthesise qualitative studies exploring sexual and gender minority migrants' experiences of health service access and utilisation.

## 4 Methods

### 4.1 Design

This was a meta‐synthesis of qualitative studies, suitable to systematically provide integrations of empirical research exploring human experiences (Sandelowski and Barroso 2007). The review was reported according to the Enhancing Transparency in Reporting the Synthesis of Qualitative Research (ENTREQ) guidelines (Tong et al. 2012) (File S1).

### 4.2 Search Methods

We conducted systematic searches in four databases indexing published scientific articles in nursing, medical, psychology and social sciences: CINAHL, PsycINFO, PubMed and Scopus. Initial searches were performed in November 2023 and updated searches were performed in July 2024. Search terms were identified through keywords in vocabulary thesauruses in the databases and through pilot searches, resulting in a combination of indexation terms (MeSH, CINAHL headings and PsycINFO descriptors) and free‐text terms with truncations to broaden the searches. Boolean operators were utilised to structure the final search strings of search terms related to SGM, forced migration and health service encounters. Searches were designed in line with the SPIDER acronym (Sample, Phenomenon of Interest, Design, Evaluation and Research type) (Cooke et al. 2012) (File S2).

The titles and abstracts of the hits in the initial searches were screened by three of the authors (CA, EN and TC), and the titles and abstracts of the hits in the updated searches were screened by the last author (TC). Hits fulfilling inclusion criteria were read as full‐text documents before the final assessment was made on inclusion. Citation screening of references in the reports included via database searches were screened by the last author (TC) to identify additional eligible reports. Reports were included regardless of the result of methodological quality appraisal. Hits and abstracts retrieved from databases were screened in Rayyan (Ouzzani et al. 2016). We included reports containing information relevant to the study aim, regardless of whether health service access and utilisation was the main focus of the study or if was presented as a part of a larger focus.

### 4.3 Inclusion and Exclusion Criteria

Inclusion criteria were (1) empirical qualitative study presented as a scientific article; (2) reports written in English; (3) reports published within 10 years; (4) based on data collected from SGM migrants and (5) contain results about the experiences of health service access and utilisation. Exclusion criteria were (1) data collected about pre‐migration or peri‐migration experiences; (2) based on data collected from migrants not presented as SGM; (3) no accessible full‐text versions of the articles; (4) quantitative and mixed‐methods studies and (5) reports published more than 10 years before the searches were conducted (Table 1).

**TABLE 1:** Inclusion and exclusion criteria.

Domain	Inclusion criteria	Exclusion criteria
Sample	Sexual and gender minority migrants	Migrants not presented as sexual and gender minority individuals
Phenomenon of interest	Access and utilisation of health services whilst residing in a host country setting	Pre‐ or peri‐migration experiences of access and utilisation of health services
Design	Individual interviews, focus group discussions or other non‐numerical qualitative designs	Numerical designs
Evaluation	Lived experiences	Observations and self‐reported ratings
Research type	Qualitative research	Quantitative and mixed‐methods research
Language	English	Non‐English
Year of publication	2013–2024	Before 2013
Publication type	Primary empirical research published as an article in a scientific journal	Conference proceedings or abstracts; book chapters; literature reviews; letters or editorials; theses and no full‐text versions available

### 4.4 Search Outcome

**FIGURE 1:**
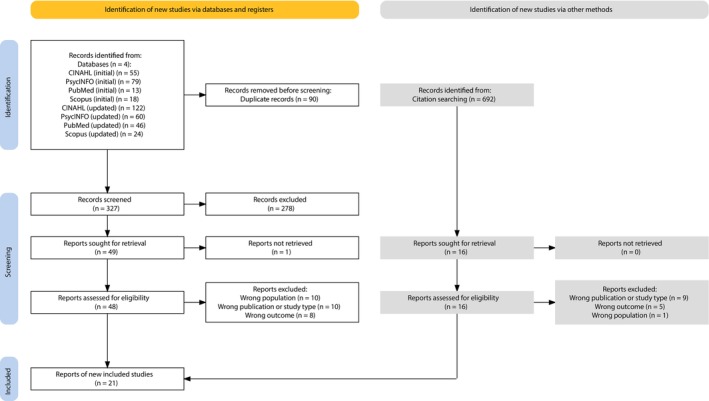
Prisma flowchart of the initial and updated searches. [Colour figure can be viewed at wileyonlinelibrary.com]

Initial and updated searches in databases resulted in 417 hits in total, of which 90 were duplicates. Of the remaining hits, 278 hits were excluded based on titles and abstracts, leaving 49 sought for retrieval of which one was not accessible. Following a full‐text assessment of the remaining reports (*n* = 48), 28 were excluded based on population (*n* = 10), study/publication type (*n* = 10) and outcome (*n* = 8). This resulted in 20 included reports identified through database searches. Additionally, a total of 692 references in the reports were screened for inclusion. Following the screening of titles, full‐text documents of 16 reports were assessed for inclusion. Fifteen were excluded based on study/publication type (*n* = 9), outcome (*n* = 5) and population (*n* = 1), resulting in 1 included report through screening of reference lists. Thus, 21 reports in total were included in this review (Figure 1 and File S3).

### 4.5 Quality Appraisal

The methodological information was extracted by the last author, according to a modified version of the JBI Qualitative Data Extraction Tool (File S4) (Lockwood et al. 2024). Ambiguous information was discussed between the first and last authors to reach a consensus. Included reports were appraised using the Critical Appraisal Skills Programme (CASP) (Critical Appraisal Skills Programme 2018) and the Joanna Briggs Institute (JBI) (Lockwood et al. 2024) checklists, which contain 10 items, respectively. The first (MG) and last (TC) authors, both senior researchers with experience in quality appraisal, appraised the included reports individually and compared their assessments to reach a consensus.

### 4.6 Data Abstraction and Synthesis

The results sections of all reports were extracted and analysed according to the process for meta‐synthesis as presented by Sandelowski and Barroso (2007). The goal of meta‐synthesis is to generate abstracted new understandings of the integrated findings from several reports. The analytic steps followed the steps in the inductive qualitative content analysis presented by Graneheim and Lundman (2004). After careful reading of all reports, meaning units were identified, condensed and attributed with a code acting as a brief statement of the content of the unit. Codes were collated into sub‐themes and themes. Frequency effect sizes (FES) were calculated for each theme and sub‐theme, defined as the percentage of reports represented in a certain sub‐category or category.

Through an iterative and collaborative process, all authors were involved in the identification of sub‐themes and themes. All authors were involved in the thematization through an iterative process. The first (MG) and last (TC) authors were responsible for the coherence and trustworthiness of the analysis, both senior researchers and nurse‐midwives with previous experience in conducting meta‐syntheses. The analysts represented a diverse group of health professionals (student nurses, nurses, midwives and psychologists), researchers (senior lecturer and associate professor), persons with lived experience of forced migration and persons of diverse sexual orientations and gender identities. The researchers engaged in close collaboration with representatives of the target population to reach in‐depth insights grounded in lived experiences.

## 5 Results

### 5.1 Methodological Characteristics of Included Studies

Table 2 and File S5 present the methodological characteristics of the included reports, which were published from 2013 to 2024 and conducted in North America (*n* = 16), Europe (*n* = 3) and Australia (*n* = 1). The majority utilised convenience sampling (*n* = 12), recruited participants through community settings (*n* = 15), collected data with semi‐structured interviews (*n* = 12) and analysed the material with thematic analysis (*n* = 11).

**TABLE 2:** Methodological characteristics of the included reports (*n* = 21).

Characteristic	Reports, *n* (%)
*Participant recruitment*	
Convenience sampling	12 (57)
Purposeful sampling	9 (43)
Snowball sampling	5 (24)
Through clinical professionals	1 (5)
Recruitment not specified	1 (5)
*Data collection*	
Semi‐structured individual interviews	17 (81)
Focus group discussions	4 (19)
Follow‐up interviews	2 (10)
Member checking sessions	1 (5)
Photovoice	1 (5)
*Region where study was conducted*	
North America	16 (76)
Europe	4 (19)
Australia	1 (5)
*Analysis procedure*	
Thematic analysis	12 (57)
Constant comparative analysis/grounded theory	4 (19)
Interpretative phenomenology analysis	2 (10)
Content analysis	1 (5)
Hermeneutic analysis	1 (5)
Participatory action research	1 (5)

**FIGURE 2:**
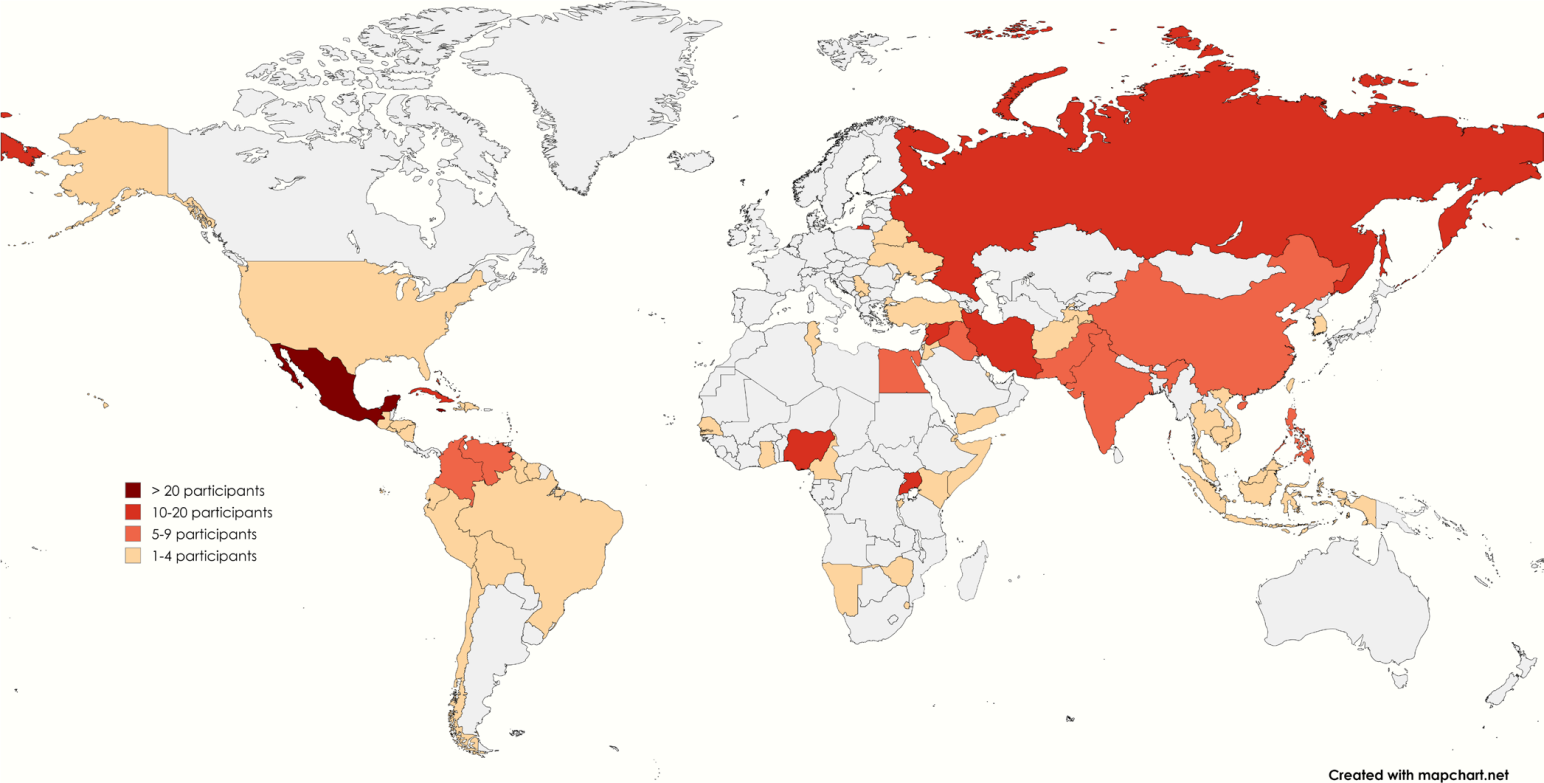
Countries of origin amongst participants. [Colour figure can be viewed at wileyonlinelibrary.com]

Table 3 presents the characteristics of the participants (*n* = 486, range: 7–92) in the included reports. Participants were between 14 and 70 years of age (range of mean or median: 24–44) and originated from 72 countries (Figure 2). The most represented countries of origin amongst participants were Mexico (*n* = 43), Jamaica (*n* = 24) and Russia (*n* = 18). Most participants were cisgender men (*n* = 235) and cisgender women (*n* = 71) self‐identifying as gay (*n* = 198), bisexual (*n* = 58) and lesbian (*n* = 42). The most represented migration status was granted, permanent residence or recognised refugee (*n* = 95). A proportion did not disclose, categorise or present their gender identity (*n* = 100), sexual orientation (*n* = 90) and migration status (*n* = 228).

**TABLE 3:** Characteristics of the participants (*n* = 486).

Characteristic	*n* (%)
*Region of origin*	
Africa	82 (17)
Asia	54 (11)
Middle East	59 (12)
South/Central America	130 (27)
Carribean	96 (20)
Europe	16 (3)
Russia	19 (4)
North America	5 (1)
*Gender identity*	
Cisgender man	235 (48)
Cisgender woman	71 (15)
Transgender woman	41 (8)
Queer, non‐binary, gender non‐conforming	12 (3)
Cisgender (unspecified)	10 (2)
Transgender (unspecified)	14 (3)
Transgender man	5 (1)
Two‐spirit	2 (0.5)
Gender not disclosed	100 (21)
*Sexual orientation*	
Gay	198 (41)
Have sex with men (unspecified)	61 (13)
Bisexual	58 (12)
Lesbian	42 (9)
Queer	18 (4)
Heterosexual	10 (2)
Pansexual	6 (1)
Other	2 (0.5)
Asexual	1 (0.2)
Sexual orientation not disclosed	90 (19)
*Migration status*	
Granted, permanent residence, recognised refugee	95 (20)
Seeking asylum, asylum applicant, asylee	33 (7)
Denied asylum, undocumented, overstay	28 (6)
Visa (Student, temporary graduate, unspecified)	29 (6)
Documented (unspecified)	16 (3)
Immigrant (unspecified)	12 (3)
Refugee or asylum seeker (unspecified)	3 (1)
Withholding of removal	2 (0.5)
Visitor, working holiday, work permit	3 (1)
Subsidiary protection, deportation ban	1 (0.2)
Migration status not presented	228 (47)

### 5.2 Quality Appraisal of Included Studies

**FIGURE 3:**
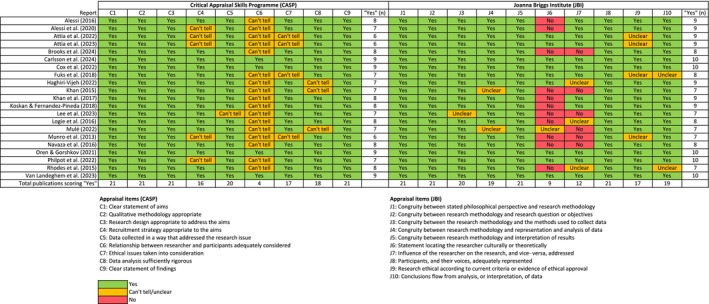
Quality appraisal results. [Colour figure can be viewed at wileyonlinelibrary.com]

Figure 3 presents the results of the methodological appraisal, which showed overall acceptable levels. All reports adhered to six or more items in CASP, with four items being adhered to by all included reports. However, only four reports adhered to the item about the relationship between the researcher and participants being adequately considered. In the JBI checklist, all reports adhered to seven or more items. Three items were adhered to by all reports, whilst only nine adhered to the item about statement locating the researcher culturally or theoretically.

### 5.3 Results of Meta‐Synthesis

The meta‐synthesis resulted in two overarching themes: *Left out of needed support: encountering barriers to accessing health services* and *Between healing and harm: the dual faces of interactions in health services* (Table 4). An expanded presentation of the results, including specific frequency effect sizes and references, is presented in File S6.

**TABLE 4:** Summary of findings with frequency effect sizes (FES).

Theme	Sub‐theme	Findings	FES (%)
Left out of Needed Support: Encountering Barriers to Accessing Health Services	Excluded From Care Based on External Barriers to Health Services	*External barriers to accessing health services included:*	
		Financial constraints	48
		Lack of information	48
		Stigma and culture	38
		Lack of health services	38
		Language requirements	19
		Living as undocumented	19
	Struggling in Silence Whilst Dealing With Internal Barriers to Health Services	*Internal barriers to accessing health services included:*	
		Fears	33
		Shame and embarrassment	29
		Prior experiences of discrimination in health services	24
Between Healing and Harm: The Dual Faces of Interactions in Health Services	Care Becomes a Burden When Facing Non‐Affirming Behaviours and Discrimination	*When interacting with health services, migrants experienced:*	
		Covert discrimination in health services	24
		Negative health impact of non‐affirming behaviours	14
		Racial discrimination	14
		Inattentiveness from health professionals	10
		Trans‐specific discrimination and non‐affirming behaviours	10
		Power imbalance favouring health professionals	5
		Lack of knowledge amongst health professionals	5
	The Power of Acceptance and Affirmation When Meeting Health Professionals	*Essential components in health services included:*	
		Personality and characteristics of health professional	33
		Adherence to confidentiality and promoting safe disclosure	29
		Openness amongst health professionals and within services	24
		Friendliness and compassion from health professionals	24
		Health professionals who validate, normalise and accept migrants	24
		Respectful communication and structures	24
	
		Ensuring that migrants' reservations, doubts and worries related to utilisation of interpreters are addressed and respected	19
		Addressing language barriers that can impact communication	14
		Health professionals who encourage migrants	10
		Competence development amongst health professionals	10
		Migrants appreciated the support received from health services	24
Migrants experienced various positive health impacts when engaging with health services	24

#### 5.3.1 Left out of Needed Support: Encountering Barriers to Accessing Health Services

The theme illustrates the lack of, and barriers to, accessing health services that migrants encounter despite needing support from health professionals. It includes two sub‐themes: *Excluded from care based on external barriers to health services* (FES 81%) (Attia et al. 2022, 2023; Brooks et al. 2024; Carlsson et al. 2024; Cox et al. 2022; Fuks et al. 2018; Haghiri‐Vijeh 2022; Kahn 2014; Kahn et al. 2018; Koskan and Fernandez‐Pineda 2018; Lee et al. 2023; Logie et al. 2016; Mulé 2022; Munro et al. 2013; Navaza et al. 2016; Oren and Gorshkov 2021; Rhodes et al. 2015; Van Landeghem et al. 2023) and *Struggling in silence whilst dealing with internal barriers to health services* (FES 57%) (Alessi et al. 2020; Brooks et al. 2024; Carlsson et al. 2024; Cox et al. 2022; Fuks et al. 2018; Haghiri‐Vijeh 2022; Kahn 2014; Kahn et al. 2018; Koskan and Fernandez‐Pineda 2018; Lee et al. 2023; Navaza et al. 2016; Philpot et al. 2022; Van Landeghem et al. 2023). Barriers to health services impacted the mental and physical health of migrants (Carlsson et al. 2024; Mulé 2022; Rhodes et al. 2015).

##### 5.3.1.1 Excluded From Care Based on External Barriers to Health Services

Migrants expressed a lack of available health services catering to their needs. A range of external barriers impaired access to health services. Stigma and cultural norms surrounding mental health treatment, living with HIV and masculinity hindered access. Financial constraints also limited access, as migrants faced high costs, lack of insurance and were ineligible for assistance. The various barriers hindering access to health services are illustrated in the following excerpt:

Participants described the multitude of barriers they face in accessing care. Emiliana noted that “the very high cost” of mental health services is a factor that limits members of their community from receiving them. For Adriana, cost and lack of insurance were also major challenges to accessing any type of mental health support. (Lee et al. 2023)

Language requirements presented additional barriers to access, including difficulties in booking appointments. Long waiting times for appointments and living as an undocumented further compounded access. A lack of information about health services was highlighted, which reduced awareness of available services and impaired access. This included information about how to access a system that was experienced as complex, how to access prevention/screening routines and the rights to health services when living as undocumented. Relatedly, a need for health information tailored to transgender identities and different language proficiencies was expressed. The following excerpt portrays the limited awareness of how to access health services:

The immigrant experience in the U.S. posed multiple challenges for ILMSM [immigrant Latino men who have sex with men] when accessing sexual health services. For example, when first arriving to the U.S., many ILMSM lacked knowledge about where and how to access services. (Brooks et al. 2024)

##### 5.3.1.2 Struggling in Silence Whilst Dealing With Internal Barriers to Health Services

Internal cognitions, feelings and fears impacted access to health services, which was influenced by the intersectional layers of the identities of migrants. Intense fears of being reported to authorities, being judged, screening discomfort and potential test results were described. The fear of being reported to authorities as a barrier to seeking care is illustrated in the following excerpt:

[Immigrant Latino men who have sex with men] were hesitant to access [HIV] services because they feared that their personal identifiable information would be reported to or shared with immigration authorities, the State government, or employers. […] also feared that accessing these services would negatively impact the immigration process and lead to their visa being revoked. (Brooks et al. 2024)

Prior experiences of discrimination in clinical settings as well as stigma and oppression carried from country of origin negatively impacted the willingness to seek health services. Shame, embarrassment and internalised stigma further hindered access. Migrants felt more inclined to access health services when professionals matched one or more of their identities. As portrayed in the following excerpt, communication with health services was impaired because of fears related to being outed:

He explained that most of the immigrants from his region chose not to discuss any health concerns with their doctors for fear of being outed and suffering other negative consequences. Therefore, Ahmed felt that LGBT [Lesbian, gay, bisexual and transgender] immigrants need to be made aware of the existence of LGBT‐friendly health care resources. (Fuks et al. 2018)

#### 5.3.2 Between Healing and Harm: The Dual Faces of Interactions in Health Services

The theme illustrates the varied experiences described in connection to utilisation of health services and during interactions with health professionals, encompassing both non‐affirming as well as affirming support. It includes the sub‐themes *Care becomes a burden when facing non‐affirming behaviours and discrimination* (FES 24%) (Brooks et al. 2024; Carlsson et al. 2024; Haghiri‐Vijeh 2022; Lee et al. 2023; Navaza et al. 2016) and *The power of acceptance and affirmation when meeting health professionals* (FES 62%) (Alessi 2016; Attia et al. 2023; Brooks et al. 2024; Carlsson et al. 2024; Cox et al. 2022; Haghiri‐Vijeh 2022; Kahn 2014; Kahn et al. 2018; Lee et al. 2023; Navaza et al. 2016; Philpot et al. 2022; Rhodes et al. 2015; Van Landeghem et al. 2023).

##### 5.3.2.1 Care Becomes a Burden When Facing Non‐Affirming Behaviours and Discrimination

Migrants encountered non‐affirming behaviours and discrimination in health services, based on both their SGM identity as well as migration and ethnicity/race. Covert and overt discrimination were outlined. Non‐affirming and discriminating encounters had a negative and traumatising impact on health and wellbeing. These instances compounded feelings of shame, hesitancy, inferiority, frustration and feeling neglected. A power imbalance between health professionals and migrants was reported, with migrants recounting being asked oppressive irrelevant questions and instances of sexual harassment, as exemplified by the following excerpt:

Participants felt that some nurses and other health professionals spoke from a position of power and privilege and asked invasive and unnecessary questions. (Haghiri‐Vijeh 2022)

Gender minority migrants encountered trans‐specific non‐affirming behaviours and discrimination in healthcare encounters, including misgendering, refusal to use correct pronouns, using names assigned at birth, degrading comments and intrusive questions. Migrants expressed unaddressed health needs based on not being listened to, understood and accepted by health professionals. They also experienced racist behaviours from professionals, which resulted in feeling mistreated and avoiding further health service utilisation. Encountering non‐affirming behaviours, such as misgendering, could add to re‐traumatization, as illustrated in the following excerpt:

Participants reported experiences of being dead named and misgendered by a wide variety of nurses and other healthcare professionals […] these brought back memories of trauma. For example, even after correcting the care provider, Ali, a trans migrant participant, was outed in the waiting room. (Haghiri‐Vijeh 2022)

Less overt forms of discrimination included not feeling welcomed and accepted, getting looks from health professionals and feeling mistreated based on language proficiency. Migrants also noted a lack of knowledge amongst health professionals regarding the health needs of SGM individuals. Moreover, they faced inattentiveness from health professionals, experiencing professionals as rushed and dismissive, which is illustrated in the following excerpt:

Some health professionals had been in a hurry during their appointments and were inattentive to their needs. Additionally, some health professionals had not addressed topics related to sexual health and were experienced as having insufficient knowledge about the health needs of sexual minorities. (Carlsson et al. 2024)

##### 5.3.2.2 The Power of Acceptance and Affirmation When Meeting Health Professionals

Migrants appreciated the support received from health services, such as help navigating the system and being offered follow‐up communication after health visits. Engaging with health services benefited their health in many ways, including relief of post‐traumatic stress symptoms, anxiety, sleeping difficulties, pain and fears. Health services improved resilience and hope, social stability, well‐being, relaxation and helped explore tensions between gender identity and faith. Whilst not all migrants felt that therapy improved their resilience, the process of interacting with mental health professionals was nevertheless widely regarded as affirming and beneficial, as illustrated in the following excerpt:

All ultimately found the process of interacting with a mental health professional to be helpful in restoring hope and mitigating distress associated with past persecution and the psychological and social impacts of flight, asylum and resettlement. (Kahn 2014)

The characteristics of health professionals were recognised as key to establishing client‐professional connections. Characteristics that could have a negative effect on connection included the personality and behaviour of professionals, when professionals did not share the migrants' SGM identity, cultural differences and when professionals originated from the same country. Competence development in intersectional identities and support for transgender individuals was emphasised, along with the importance of respectful and affirming health services. Participants appreciated compassionate, friendly and encouraging health professionals who accepted them and validated their experiences. They needed a safe and welcoming space where they could discuss their sexual health in a non‐judgmental setting. Openness was crucial, with the need for open‐minded professionals who were willing to learn from them, respected their faith and enabled free discussion of questions:

The participants felt heard and accepted when nurses and other healthcare professionals listened attentively, respected them, were aware of their past traumatic experiences, and provided compassionate, caring, kind, and nonjudgmental care. (Haghiri‐Vijeh 2022)

Migrants were concerned about disclosure and confidentiality when interacting with health services, worrying about the consequences if sensitive data was leaked from health journals. Heteronormative assumptions amongst professionals and cultural stigma hindered disclosure. Migrants worried how professionals would react if they disclosed their identity. To avoid visibility and feel comfortable, some decided to conceal their SGM identity and preferred specialised health services. Specifically, waiting rooms and receptions were spaces where their identity could be exposed, requiring sensitivity from professionals, as portrayed in the following excerpt:

There were concerns that being seen in the waiting room might arouse assumptions from other patients about their HIV status, thus implicitly contravening confidentiality. […] His concerns about waiting room confidentiality were drawn from his experiences in his country of origin, where presence in waiting rooms in sexual health clinics could attract gossip from others in the community. (Philpot et al. 2022)

Language barriers made it difficult to express health concerns, with migrants stating a need for adequate interpreter services. However, various reservations and doubts were articulated regarding the utilisation of interpreters. This included feeling uncomfortable and embarrassed when several people were in the room, fearing how the interpreter would react and the potential risk of sensitive information being spread through the interpreter. Consequently, some preferred telephonic interpretation to remain anonymous. The sensitivity concerns related to interpreter services are portrayed in the following excerpt:

Participants felt that the translation services provided were inadequate or created situations where they were either uncomfortable or embarrassed discussing their sexual behaviors with multiple individuals. (Brooks et al. 2024)

## 6 Discussion

The findings illustrate barriers to accessing health services, as well as the importance of affirming support from health professionals. Our review adds to the existing literature of reviews investigating the health of SGM migrants (Alessi et al. 2021; Gottvall, Brunell, Eldebo, Johansson Metso, et al. 2023; Yarwood et al. 2022), by focusing on specific experiences related to health service access and utilisation. The results align with the intersectional minority stress model, highlighting the impact of oppression against people who have a sexual orientation and gender identity extending beyond societal norms (Rivas‐Koehl et al. 2023).

Health disparities continue to be a pressing concern throughout the world, specifically those impacting the health of racial‐ethnic minorities and SGM populations (Mongelli et al. 2020). There is evidence that the health and well‐being of the wider population of migrants are impacted by a range of barriers (Nowak et al. 2022; Parajuli and Horey 2020), which is confirmed by our findings. This review identified specific barriers to health service access, including social stigma, cultural norms and fears of encountering judgmental behaviours. Echoing our findings, previous research outlines that SGM populations avoid seeking health services because of prior experiences of discrimination, internalised stigma and minority stress (Kuzma et al. 2019; Thomas et al. 2024). The findings point to the importance of applying an intersectional perspective (Rivas‐Koehl et al. 2023) when addressing barriers to accessing health services. Despite efforts to achieve accessible health services for all people, our findings point out that these migrants are at risk of impaired access and utilisation of health services based on multiple minority identities. We urge health services and decision‐makers to break down these barriers. More research is needed to understand how to achieve accessible health services for all, including this marginalised population.

According to the minority stress model, SGM individuals face chronic stress and health burdens due to social stigmatisation (Rivas‐Koehl et al. 2023). Indeed, exposure to structural stigma can induce minority stress and mental health burdens in the host country (van der Star et al. 2021). Ensuring affirming and trauma‐informed support for SGM (Pachankis and Bränström 2018; Sherman et al. 2023) and migrant populations (Shi et al. 2021) is essential. However, our review identified that non‐affirming behaviours and discrimination were encountered when interacting with health services. Studies have repeatedly shown that patients encounter racism in health services, contributing to a lack of trust and delays in seeking support or treatment (Hamed et al. 2022). Similarly, SGM individuals face stigma, refusal of healthcare and verbal or physical abuse from health professionals (Ayhan et al. 2020). Research investigating discrimination in health services often lacks intersectional approaches taking into consideration several interacting axes of discrimination (Merz et al. 2024). Our review adds to the existing literature by providing valuable insights into the intersectional experiences of discrimination and non‐affirming behaviours.

Validation and normalisation were identified as key features in clinical encounters. A safe encounter increased the willingness to disclose their sexuality and gender identity. There is an acknowledged need to improve the competence amongst health professionals regarding the health needs of SGM individuals (Bird et al. 2024; Sherman et al. 2023) and migrants (Olaussen and Renzaho 2016). Strategies to enhance competence in health services span from individual to organisational level, involving different aspects such as continuity of care, audiovisual materials, interpreter services and competence training (Handtke et al. 2019). Our review highlights various components in need of consideration related to implementing strategies like these. Specifically, providing competence development for nurses, about how to provide safe and affirming health services to SGM individuals, has the potential to improve knowledge and enhance health equity (Kuzma et al. 2019; Traister 2020). Moreover, students and lecturers in nursing education call attention to the need for more educational content about the health needs of SGM migrants (Gottvall, Brunell, Eldebo, Kissiti, et al. 2023). This further underscores the importance of cultural safety training for health professionals and the need for targeted culturally sensitive health promotion (Baptiste‐Roberts et al. 2017) for this population. We encourage more research investigating strategies to address clinical safety and cultural sensitivity through the training of health professionals.

## 7 Limitations of the Study

There are limitations to this review. Whilst we argue that our searches were comprehensive and resulted in many reports included, it is nevertheless possible that we unintentionally failed to identify or dismiss reports fulfilling inclusion criteria. Using more databases, additional search terms and wider inclusion criteria could have resulted in additional reports containing valuable information. The quality appraisal was conducted by two senior researchers and indicated acceptable quality. If more assessors had been engaged, it would have involved less risk of bias. The tools used for methodological appraisal are commonly used in qualitative evidence syntheses and involve a range of quality criteria (Majid and Vanstone 2018). Whilst the studies received high appraisals many lacked sufficient details about aspects related to reflexivity, which is widely recognised as an important part of qualitative research (Patton 2015). However, the inclusion of reflexivity appraisal criteria has been criticised based on challenges related to subjectivity and the difficulties in assessing it accurately (Majid and Vanstone 2018). It is probable that researchers involved in the included studies engaged in reflexive activities, but that these activities were not described enough in the reports to achieve a high quality appraisal.

Whilst there was a wide variation of countries of origin represented, most of the included reports were conducted in North America. Further, few participants self‐identified as other than cisgender gay men. Readers should note that we did not examine differences depending on legal migration status, and migration status was not presented for almost half of the participants. It is possible that there are specific experiences amongst migrants with certain statuses, such as undocumented migrants, which need further exploration. Moreover, there is a need for research on the specific experiences of bisexual people, lesbian women and transgender/gender non‐conforming migrants. We encourage more research conducted in other regions and including a wider diversity of participants. Lastly, the synthesis of findings may have been influenced by interpreter bias. To approach the data from diverse perspectives, experts lived experience closely collaborated with the researchers and a clinical psychologist throughout the analysis. We argue that our collaborative analytic process promoted democratised in‐depth understanding grounded in lived experience.

## 8 Conclusions

Sexual and gender minority migrants face a range of barriers when needing health services, contributing to unmet health needs and health disparities. Whilst some barriers are shared with the wider population of migrants, others are closely tied to the minority identity of being both a migrant and identifying as a sexual and gender minority. When interacting with health services and health professionals, migrants experience intersectional discrimination and non‐affirming behaviours based on their ethnicity, migration status, gender identity, gender expression and sexual orientation. Ensuring safety is a key aspect of achieving high‐quality and accessible health services.

## 9 Relevance to Clinical Practice

Nurses and other health professionals need to address the many barriers that impair access to health services for migrants. Importantly, fears of encountering judgmental behaviours and discrimination can contribute to the avoidance of health services. Thus, nurses and other health professionals should apply a clinical approach taking into consideration minority stress layered by multiple intersecting identities. The insights from this review are relevant for clinical practice as they underscore the necessity for health professionals to adopt culturally sensitive and affirming care approaches. This includes deepening their awareness of the specific health needs and challenges faced by these migrants. Ensuring safety through affirming support is key to achieving high‐quality and accessible health services for sexual and gender minority migrants. Nurses and other health professionals need to carefully consider intersectional layers related to sensitivity and safety. Open, friendly, validating, respectful and encouraging communication is essential. By creating an environment that is inclusive and respectful of diverse identities, health professionals may help alleviate fears and build trust.

## Author Contributions

**Maria Gottvall:** conceptualization; meta‐synthesis analysis; funding acquisition; quality appraisal; writing – original draft. **Osszián Péter‐Szabó:** meta‐synthesis analysis; writing – original draft. **Rummage Isaac:** meta‐synthesis analysis; writing – original draft. **Christoffer Aav:** citation screening; database screening; meta‐synthesis analysis; writing – review and editing. **Erik Norgren:** citation screening; database screening; meta‐synthesis analysis; writing – review and editing. **Tommy Carlsson:** conceptualization; citation screening; database screening; meta‐synthesis analysis; funding acquisition; project administration; supervision; quality appraisal; writing – original draft.

## Conflicts of Interest

The authors declare no conflicts of interest.

## Supporting information


File S1.

File S2.

File S3.

File S4.

File S5.

File S6.

## Data Availability

Data sharing is not applicable to this article as no new data were created or analyzed in this study.
