# Supplementary material for: Sexual and Gender Minority Migrants' Experiences of Health Service Access and Utilisation: A Qualitative Meta‐Synthesis
Source: J Clin Nurs. 2025 Feb 14;34(10):4448–61. doi: 10.1111/jocn.17683 (PMC12409289; doi:10.1111/jocn.17683)
Supplement: Supplementary file 2 — File S2. [file JOCN-34-4448-s004.pdf]

Supplementary File 2. Search strings for the searches in the databases.

SPIDER	Focus	Search strings
Sample	(1) Forced migrants self-identifying as sexual and gender minorities and (2) health professionals supporting them	(("Sexual and Gender Minorities") OR (queer*) OR (homosexual*) OR (”sexual minorit*”) OR (”sexual orientation*”) OR (”gender minorit*”) OR (lesbian*) OR (gay) OR (bisexual*) OR (”same sex couple*”) OR (”same sex relation*”) OR (”women loving women”) OR (”men who have sex with men”) OR (”women who have sex with women”) OR (Lesbigay) OR (”Non-Heterosex*”) OR (”Non heterosex*”) OR (GLB) OR (GLBT) OR (GLBTQ) OR (LGB) OR (LGBT) OR (LGBTQ) OR (LGBTQI) OR (LGBTQIA) OR (transgender*) OR (transsexual*) OR (”gender dysphoria”) OR (”gender nonbinary”) OR (”nonbinary gender”) OR (nonbinary) OR (”non binary”) OR (transvesti*) OR (crossgender) OR (”gender change”) OR (”gender transform*”) OR (”gender transition”) OR (genderqueer) OR (”trans female*”) OR (”trans male*”) OR (”trans man”) OR (”trans men”) OR (”trans people”) OR (”trans person”) OR (”trans woman”) OR (”trans women”) OR (”gender identity disorder*”) OR (bicurious) OR (intersex*) OR (Asexual*) OR (”Two- spirit*”) OR (Pansex*) OR (”Gender queer”) OR (Agender*) OR (Bigender*) OR (Pangender*) OR (Omnisexual*) OR (”Gender variant*”) OR (”Gender fluid”) OR (”SOGIE”) OR (”Diverse sexual orientation*”) OR (”Diverse gender*”)) AND (("Emigrants and Immigrants"[Mesh]) OR (”Emigration and Immigration”) OR (”Refugees”[Mesh]) OR (Refugee*) OR (”War-Related Injuries”[Mesh]) OR (Newcomer*) OR (Settler*) OR (Noncitizen*) OR (”Forced migrant*”) OR (Incomer*) OR (Incoming*) OR (Foreign*) OR (Asylum*) OR (Undocumented))
Phenomenon of Interest	Clinical support and interactions between sexual minority forced migrants and health professionals	((”Interpersonal Relations”) OR (”Professional-Patient Relations”) OR (“Patient-Centered Care”) OR (”Health Personnel”) OR (Nurse*) OR (”Mental health professional*”) OR (Psychologist*) OR (Midwi*) OR (Doctor*) OR (Physician*) OR (Clinician*) OR (Therapist*) OR (Counselor*) OR (Provider*) OR (Professional*) OR (“Health services”[Mesh]))
Design	Individual interviews and/or focus group discussions	((Interview*) OR (”Focus group*”) OR (“Discussion*”) OR (“Narrative*”) OR (”Qualitative research”) OR (“Qualitative*”) OR (“Phenomen*”) OR (“Grounded theor*”) OR (“Ethnograph*”) (“Content analys*”) OR (“Thematic analys*”))
Evaluation	Lived experiences	((“Experience*”) OR (“Lived experience*”) OR (“Perspective*”))
Research type	Qualitative research	((Interview*) OR (”Focus group*”) OR (“Discussion*”) OR (“Narrative*”) OR (”Qualitative research”) OR (“Qualitative*”) OR (“Phenomen*”) OR (“Grounded theor*”) OR (“Ethnograph*”) (“Content analys*”) OR (“Thematic analys*”))
