# Supplementary material for: Sexual and Gender Minority Migrants' Experiences of Health Service Access and Utilisation: A Qualitative Meta‐Synthesis
Source: J Clin Nurs. 2025 Feb 14;34(10):4448–61. doi: 10.1111/jocn.17683 (PMC12409289; doi:10.1111/jocn.17683)
Supplement: Supplementary file 3 — File S3. [file JOCN-34-4448-s001.pdf]

Supplementary File 3. Outcome of searches in databases.

Initial searches

Database: Pubmed
Date: 2023-11-08
Hits: 13
Complete search string: (("Sexual and Gender Minorities"[Mesh]) OR (queer*) OR (homosexual*) OR (”sexual minorit*”) OR (”sexual orientation*”) OR (”gender minorit*”) OR (lesbian*) OR (gay) OR (bisexual*) OR (”same sex couple*”) OR (”same sex relation*”) OR (”women loving women”) OR (”men who have sex with men”) OR (”women who have sex with women”) OR (Lesbigay) OR (”Non-Heterosex*”) OR (”Non heterosex*”) OR (GLB) OR (GLBT) OR (GLBTQ) OR (LGB) OR (LGBT) OR (LGBTQ) OR (LGBTQI) OR (LGBTQIA) OR (transgender*) OR (transsexual*) OR (”gender dysphoria”) OR (”gender nonbinary”) OR (”nonbinary gender”) OR (nonbinary) OR (”non binary”) OR (transvesti*) OR (crossgender) OR (”gender change”) OR (”gender transform*”) OR (”gender transition”) OR (genderqueer) OR (”trans female*”) OR (”trans male*”) OR (”trans man”) OR (”trans men”) OR (”trans people”) OR (”trans person”) OR (”trans woman”) OR (”trans women”) OR (”gender identity disorder*”) OR (bicurious) OR (intersex*) OR (Asexual*) OR (”Two- spirit*”) OR (Pansex*) OR (”Gender queer”) OR (Agender*) OR (Bigender*) OR (Pangender*) OR (Omnisexual*) OR (”Gender variant*”) OR (”Gender fluid”) OR (”SOGIE”) OR (”Diverse sexual orientation*”) OR (”Diverse gender*”)) AND (("Emigrants and Immigrants"[Mesh]) OR (”Emigration and Immigration”[Mesh]) OR (”Refugees”[Mesh]) OR (Refugee*) OR (”War-Related Injuries”[Mesh]) OR (Newcomer*) OR (Settler*) OR (Noncitizen*) OR (”Forced migrant*”) OR (Incomer*) OR (Incoming*) OR (Foreign*) OR (Asylum*) OR (Undocumented)) AND ((”Interpersonal Relations” [Mesh]) OR (”Professional-Patient Relations”[Mesh]) OR (“Patient-Centered Care”[Mesh]) OR (”Health Personnel” [Mesh]) OR (Nurse*) OR (”Mental health professional*”) OR (Psychologist*) OR (Midwi*) OR (Doctor*) OR (Physician*) OR (Clinician*) OR (Therapist*) OR (Counselor*) OR (Provider*) OR (Professional*) OR (“Health services”[Mesh])) AND ((“Experience*”) OR (“Lived experience*”) OR (“Perspective*”)) AND ((Interview*) OR (”Focus group*”) OR (“Discussion*”) OR (“Narrative*”) OR (”Qualitative research” [Mesh]) OR (“Qualitative*”) OR (“Phenomen*”) OR (“Grounded theor*”) OR (“Ethnograph*”) (“Content analys*”) OR (“Thematic analys*”))

Database: CINAHL
Date: 2023-11-08
Hits: 55
Complete search string: ((MH "Sexual and Gender Minorities+") OR (queer*) OR (homosexual*) OR (”sexual minorit*”) OR (”sexual orientation*”) OR (”gender minorit*”) OR (lesbian*) OR (gay) OR (bisexual*) OR (”same sex couple*”) OR (”same sex relation*”) OR (”women loving women”) OR (”men who have sex with men”) OR (”women who have sex with women”) OR (Lesbigay) OR (”Non-Heterosex*”) OR (”Non heterosex*”) OR (GLB) OR (GLBT) OR (GLBTQ) OR (LGB) OR (LGBT) OR (LGBTQ) OR (LGBTQI) OR (LGBTQIA) OR (transgender*) OR (transsexual*) OR (”gender dysphoria”) OR (”gender nonbinary”) OR (”nonbinary gender”) OR (nonbinary) OR (”non binary”) OR (transvesti*) OR (crossgender) OR (”gender change”) OR (”gender transform*”) OR (”gender transition”) OR (genderqueer) OR (”trans female*”) OR (”trans male*”) OR (”trans man”) OR (”trans men”) OR (”trans

people”) OR (”trans person”) OR (”trans woman”) OR (”trans women”) OR (”gender identity disorder*”) OR (bicurious) OR (intersex*) OR (Asexual*) OR (”Two- spirit*”) OR (Pansex*) OR (”Gender queer”) OR (Agender*) OR (Bigender*) OR (Pangender*) OR (Omnisexual*) OR (”Gender variant*”) OR (”Gender fluid”) OR (”SOGIE”) OR (”Diverse sexual orientation*”) OR (”Diverse gender*”)) AND ((MH "Immigrants+") OR (MM "Undocumented Immigrants") OR ("Emigrants and Immigrants") OR (”Emigration and Immigration”) OR (MH "Refugees+") OR (Refugee*) OR (”War-Related Injuries”) OR (Newcomer*) OR (Settler*) OR (Noncitizen*) OR (”Forced migrant*”) OR (Incomer*) OR (Incoming*) OR (Foreign*) OR (Asylum*) OR (Undocumented)) AND ((MH "Interpersonal Relations+") OR (MH "Professional-Patient Relations+") OR (MM "Patient Centered Care") OR (MH "Health Personnel+") OR (Nurse*) OR (”Mental health professional*”) OR (Psychologist*) OR (Midwi*) OR (Doctor*) OR (Physician*) OR (Clinician*) OR (Therapist*) OR (Counselor*) OR (Provider*) OR (Professional*) OR (MH "Health Services+")) AND ((“Experience*”) OR (“Lived experience*”) OR (“Perspective*”)) AND ((Interview*) OR (”Focus group*”) OR (“Discussion*”) OR (“Narrative*”) OR (MH "Qualitative Studies+") OR (Qualitative research”) OR (“Qualitative*”) OR (“Phenomen*”) OR (“Grounded theor*”) OR (“Ethnograph*”) (“Content analys*”) OR (“Thematic analys*”))

Database: PsycINFO
Date: 2023-11-08
Hits: 79
Complete search string: ((DE "Sexual Orientation") OR (queer*) OR (homosexual*) OR (”sexual minorit*”) OR (”sexual orientation*”) OR (”gender minorit*”) OR (lesbian*) OR (gay) OR (bisexual*) OR (”same sex couple*”) OR (”same sex relation*”) OR (”women loving women”) OR (”men who have sex with men”) OR (”women who have sex with women”) OR (Lesbigay) OR (”Non-Heterosex*”) OR (”Non heterosex*”) OR (GLB) OR (GLBT) OR (GLBTQ) OR (LGB) OR (LGBT) OR (LGBTQ) OR (LGBTQI) OR (LGBTQIA) OR (transgender*) OR (transsexual*) OR (”gender dysphoria”) OR (”gender nonbinary”) OR (”nonbinary gender”) OR (nonbinary) OR (”non binary”) OR (transvesti*) OR (crossgender) OR (”gender change”) OR (”gender transform*”) OR (”gender transition”) OR (genderqueer) OR (”trans female*”) OR (”trans male*”) OR (”trans man”) OR (”trans men”) OR (”trans people”) OR (”trans person”) OR (”trans woman”) OR (”trans women”) OR (”gender identity disorder*”) OR (bicurious) OR (intersex*) OR (Asexual*) OR (”Two- spirit*”) OR (Pansex*) OR (”Gender queer”) OR (Agender*) OR (Bigender*) OR (Pangender*) OR (Omnisexual*) OR (”Gender variant*”) OR (”Gender fluid”) OR (”SOGIE”) OR (”Diverse sexual orientation*”) OR (”Diverse gender*”)) AND ((DE "Immigration") OR (DE “Undocumented Immigration”) OR ("Emigrants and Immigrants") OR (”Emigration and Immigration”) OR (MM "Refugees") OR (Refugee*) OR (”War-Related Injuries”) OR (Newcomer*) OR (Settler*) OR (Noncitizen*) OR (”Forced migrant*”) OR (Incomer*) OR (Incoming*) OR (Foreign*) OR (Asylum*) OR (Undocumented)) AND ((DE "Interpersonal Relationships") OR ("Professional-Patient Relation*") OR (MM "Patient Centered Care") OR (DE "Health Personnel") OR (DE "Allied Health Personnel") OR (DE "Caregivers") OR (DE "Medical Personnel") OR (DE "Mental Health Personnel") OR (Nurse*) OR (”Mental health professional*”) OR (Psychologist*) OR (Midwi*) OR (Doctor*) OR (Physician*) OR (Clinician*) OR (Therapist*) OR (Counselor*) OR (Provider*) OR (Professional*) OR ("Health Service*")) AND ((“Experience*”) OR (“Lived experience*”) OR (“Perspective*”)) AND ((Interview*) OR (”Focus group*”) OR (“Discussion*”) OR (“Narrative*”) OR ("Qualitative Stud*") OR (Qualitative research”) OR (“Qualitative*”) OR

(“Phenomen*”) OR (“Grounded theor*”) OR (“Ethnograph*”) (“Content analys*”) OR (“Thematic analys*”))

Database: Scopus
Date: 2023-11-08
Hits: 18
Complete search string: (("Sexual and Gender Minorit*”) OR (queer*) OR (homosexual*) OR (”sexual minorit*”) OR (”sexual orientation*”) OR (”gender minorit*”) OR (lesbian*) OR (gay) OR (bisexual*) OR (”same sex couple*”) OR (”same sex relation*”) OR (”women loving women”) OR (”men who have sex with men”) OR (”women who have sex with women”) OR (Lesbigay) OR (”Non-Heterosex*”) OR (”Non heterosex*”) OR (GLB) OR (GLBT) OR (GLBTQ) OR (LGB) OR (LGBT) OR (LGBTQ) OR (LGBTQI) OR (LGBTQIA) OR (transgender*) OR (transsexual*) OR (”gender dysphoria”) OR (”gender nonbinary”) OR (”nonbinary gender”) OR (nonbinary) OR (”non binary”) OR (transvesti*) OR (crossgender) OR (”gender change”) OR (”gender transform*”) OR (”gender transition”) OR (genderqueer) OR (”trans female*”) OR (”trans male*”) OR (”trans man”) OR (”trans men”) OR (”trans people”) OR (”trans person”) OR (”trans woman”) OR (”trans women”) OR (”gender identity disorder*”) OR (bicurious) OR (intersex*) OR (Asexual*) OR (”Two- spirit*”) OR (Pansex*) OR (”Gender queer”) OR (Agender*) OR (Bigender*) OR (Pangender*) OR (Omnisexual*) OR (”Gender variant*”) OR (”Gender fluid”) OR (”SOGIE”) OR (”Diverse sexual orientation*”) OR (”Diverse gender*”)) AND (("Emigrants and Immigrants") OR (”Emigration and Immigration”) OR (”Refugees”) OR (Refugee*) OR (”War-Related Injuries”) OR (Newcomer*) OR (Settler*) OR (Noncitizen*) OR (”Forced migrant*”) OR (Incomer*) OR (Incoming*) OR (Foreign*) OR (Asylum*) OR (Undocumented)) AND ((”Interpersonal Relations”) OR (”Professional-Patient Relations”) OR (“Patient-Centered Care”) OR (”Health Personnel”) OR (Nurse*) OR (”Mental health professional*”) OR (Psychologist*) OR (Midwi*) OR (Doctor*) OR (Physician*) OR (Clinician*) OR (Therapist*) OR (Counselor*) OR (Provider*) OR (Professional*) OR (“Health service*”)) AND ((“Experience*”) OR (“Lived experience*”) OR (“Perspective*”)) AND ((Interview*) OR (”Focus group*”) OR (“Discussion*”) OR (“Narrative*”) OR (”Qualitative research”) OR (“Qualitative*”) OR (“Phenomen*”) OR (“Grounded theor*”) OR (“Ethnograph*”) (“Content analys*”) OR (“Thematic analys*”))

Updated searches

Database: PubMed
Date: 2024-07-20
Hits: 46
Complete search string: (("Sexual and Gender Minorities"[Mesh]) OR (queer*[tiab]) OR (homosexual*[tiab]) OR ("sexual minorit*"[tiab]) OR ("sexual orientation*"[tiab]) OR ("gender minorit*"[tiab]) OR (lesbian*[tiab]) OR (gay[tiab]) OR (bisexual*[tiab]) OR ("same sex couple*"[tiab]) OR ("same sex relation*"[tiab]) OR ("women loving women"[tiab]) OR ("men who have sex with men"[tiab]) OR ("women who have sex with women"[tiab]) OR (Lesbigay[tiab]) OR ("Non-Heterosex*"[tiab]) OR ("Non heterosex*"[tiab]) OR (GLB[tiab]) OR (GLBT[tiab]) OR (GLBTQ[tiab]) OR (LGB[tiab]) OR (LGBT[tiab]) OR (LGBTQ[tiab]) OR (LGBTQI[tiab]) OR (LGBTQIA[tiab]) OR (transgender*[tiab]) OR (transsexual*[tiab]) OR ("gender dysphoria"[tiab]) OR ("gender nonbinary"[tiab]) OR ("nonbinary gender"[tiab]) OR (nonbinary[tiab]) OR ("non binary"[tiab]) OR (transvesti*[tiab]) OR (crossgender[tiab]) OR

("gender change"[tiab]) OR ("gender transform*"[tiab]) OR ("gender transition"[tiab]) OR (genderqueer[tiab]) OR ("trans female*"[tiab]) OR ("trans male*"[tiab]) OR ("trans man"[tiab]) OR ("trans men"[tiab]) OR ("trans people"[tiab]) OR ("trans person"[tiab]) OR ("trans woman"[tiab]) OR ("trans women"[tiab]) OR ("gender identity disorder*"[tiab]) OR (bicurious[tiab]) OR (intersex*[tiab]) OR (Asexual*[tiab]) OR ("Two- spirit*"[tiab]) OR (Pansex*[tiab]) OR ("Gender queer"[tiab]) OR (Agender*[tiab]) OR (Bigender*[tiab]) OR (Pangender*[tiab]) OR (Omnisexual*[tiab]) OR ("Gender variant*"[tiab]) OR ("Gender fluid"[tiab]) OR ("SOGIE"[tiab]) OR ("Diverse sexual orientation*"[tiab]) OR ("Diverse gender*"[tiab])) AND (("Emigrants and Immigrants"[Mesh]) OR ("Emigration and Immigration"[Mesh]) OR ("Refugees"[Mesh]) OR (Refugee*[tiab]) OR ("War-Related Injuries"[Mesh]) OR (Newcomer*[tiab]) OR (Settler*[tiab]) OR (Noncitizen*[tiab]) OR ("Forced migrant*"[tiab]) OR (Incomer*[tiab]) OR (Incoming*[tiab]) OR (Foreign*[tiab]) OR (Asylum*[tiab]) OR (Undocumented[tiab])) AND (("Interpersonal Relations" [Mesh]) OR ("Professional-Patient Relations"[Mesh]) OR ("Patient-Centered Care"[Mesh]) OR ("Health Personnel"[Mesh]) OR (Nurse*[tiab]) OR ("Mental health professional*"[tiab]) OR (Psychologist*[tiab]) OR (Midwi*[tiab]) OR (Doctor*[tiab]) OR (Physician*[tiab]) OR (Clinician*[tiab]) OR (Therapist*[tiab]) OR (Counselor*[tiab]) OR (Provider*[tiab]) OR (Professional*[tiab]) OR ("Health services"[Mesh])) AND (("Experience*"[tiab]) OR ("Lived experience*"[tiab]) OR ("Perspective*"[tiab])) AND ((Interview*[tiab]) OR ("Focus group*"[tiab]) OR ("Discussion*"[tiab]) OR ("Narrative*"[tiab]) OR ("Qualitative research"[Mesh]) OR (“Qualitative*”[tiab]) OR ("Phenomen*"[tiab]) OR ("Grounded theor*"[tiab]) OR ("Ethnograph*"[tiab]) OR ("Content analys*"[tiab]) OR ("Thematic analys*"[tiab])))

Database: CINAHL
Date: 2024-07-20
Hits: 122
Complete search string: ((MH "Sexual and Gender Minorities+") OR (TX queer*) OR (TX homosexual*) OR (TX ”sexual minorit*”) OR (TX ”sexual orientation*”) OR (TX ”gender minorit*”) OR (TX lesbian*) OR (TX gay) OR (TX bisexual*) OR (TX ”same sex couple*”) OR (TX ”same sex relation*”) OR (TX ”women loving women”) OR (TX ”men who have sex with men”) OR (TX ”women who have sex with women”) OR (TX Lesbigay) OR (TX ”Non-Heterosex*”) OR (TX ”Non heterosex*”) OR (TX GLB) OR (TX GLBT) OR (TX GLBTQ) OR (TX LGB) OR (TX LGBT) OR (TX LGBTQ) OR (TX LGBTQI) OR (TX LGBTQIA) OR (TX transgender*) OR (TX transsexual*) OR (TX ”gender dysphoria”) OR (TX ”gender nonbinary”) OR (TX ”nonbinary gender”) OR (TX nonbinary) OR (TX ”non binary”) OR (TX transvesti*) OR (TX crossgender) OR (TX ”gender change”) OR (TX ”gender transform*”) OR (TX ”gender transition”) OR (TX genderqueer) OR (TX ”trans female*”) OR (TX ”trans male*”) OR (TX ”trans man”) OR (TX ”trans men”) OR (TX ”trans people”) OR (TX ”trans person”) OR (TX ”trans woman”) OR (TX ”trans women”) OR (TX ”gender identity disorder*”) OR (TX bicurious) OR (TX intersex*) OR (TX Asexual*) OR (TX ”Two- spirit*”) OR (TX Pansex*) OR (TX ”Gender queer”) OR (TX Agender*) OR (TX Bigender*) OR (TX Pangender*) OR (TX Omnisexual*) OR (TX ”Gender variant*”) OR (TX ”Gender fluid”) OR (TX ”SOGIE”) OR (TX ”Diverse sexual orientation*”) OR (TX ”Diverse gender*”)) AND ((MH "Immigrants+") OR (MM "Undocumented Immigrants") OR (TX "Emigrants and Immigrants") OR (TX ”Emigration and Immigration”) OR (MH "Refugees+") OR (TX Refugee*) OR (TX ”War-Related Injuries”) OR (TX Newcomer*) OR (TX Settler*) OR (TX Noncitizen*) OR (TX ”Forced migrant*”) OR (TX Incomer*) OR (TX

Incoming*) OR (TX Foreign*) OR (TX Asylum*) OR (TX Undocumented)) AND ((MH "Interpersonal Relations+") OR (MH "Professional-Patient Relations+") OR (MM "Patient Centered Care") OR (MH "Health Personnel+") OR (TX Nurse*) OR (TX ”Mental health professional*”) OR (TX Psychologist*) OR (TX Midwi*) OR (TX Doctor*) OR (TX Physician*) OR (TX Clinician*) OR (TX Therapist*) OR (TX Counselor*) OR (TX Provider*) OR (TX Professional*) OR (MH "Health Services+")) AND ((TX “Experience*”) OR (TX “Lived experience*”) OR (TX “Perspective*”)) AND ((TX Interview*) OR (TX ”Focus group*”) OR (TX “Discussion*”) OR (TX “Narrative*”) OR (MH "Qualitative Studies+") OR (TX "Qualitative research”) OR (TX “Qualitative*”) OR (TX “Phenomen*”) OR (TX “Grounded theor*”) OR (TX “Ethnograph*”) OR (TX “Content analys*”) OR (TX “Thematic analys*”))

Database: PsycINFO
Date: 2024-07-20
Hits: 60
Complete search string: ((DE "Sexual Orientation") OR ((AB queer*) OR (TI queer*)) OR ((AB homosexual*) OR (TI homosexual*)) OR ((AB "sexual minorit*") OR (TI "sexual minorit*")) OR ((AB "sexual orientation*") OR (TI "sexual orientation*")) OR ((AB "gender minorit*") OR (TI "gender minorit*")) OR ((AB lesbian*) OR (TI lesbian*)) OR ((AB gay) OR (TI gay)) OR ((AB bisexual*) OR (TI bisexual*)) OR ((AB "same sex couple*") OR (TI "same sex couple*")) OR ((AB "same sex relation*") OR (TI "same sex relation*")) OR ((AB "women loving women") OR (TI "women loving women")) OR ((AB "men who have sex with men") OR (TI "men who have sex with men")) OR ((AB "women who have sex with women") OR (TI "women who have sex with women")) OR ((AB Lesbigay) OR (TI Lesbigay)) OR ((AB "Non-Heterosex*") OR (TI "Non-Heterosex*")) OR ((AB "Non heterosex*") OR (TI "Non heterosex*")) OR ((AB GLB) OR (TI GLB)) OR ((AB GLBT) OR (TI GLBT)) OR ((AB GLBTQ) OR (TI GLBTQ)) OR ((AB LGB) OR (TI LGB)) OR ((AB LGBT) OR (TI LGBT)) OR ((AB LGBTQ) OR (TI LGBTQ)) OR ((AB LGBTQI) OR (TI LGBTQI)) OR ((AB LGBTQIA) OR (TI LGBTQIA)) OR ((AB transgender*) OR (TI transgender*)) OR ((AB transsexual*) OR (TI transsexual*)) OR ((AB "gender dysphoria") OR (TI "gender dysphoria")) OR ((AB "gender nonbinary") OR (TI "gender nonbinary")) OR ((AB "nonbinary gender") OR (TI "nonbinary gender")) OR ((AB nonbinary) OR (TI nonbinary)) OR ((AB "non binary") OR (TI "non binary")) OR ((AB transvesti*) OR (TI transvesti*)) OR ((AB crossgender) OR (TI crossgender)) OR ((AB "gender change") OR (TI "gender change")) OR ((AB "gender transform*") OR (TI "gender transform*")) OR ((AB "gender transition") OR (TI "gender transition")) OR ((AB genderqueer) OR (TI genderqueer)) OR ((AB "trans female*") OR (TI "trans female*")) OR ((AB "trans male*") OR (TI "trans male*")) OR ((AB "trans man") OR (TI "trans man")) OR ((AB "trans men") OR (TI "trans men")) OR ((AB "trans people") OR (TI "trans people")) OR ((AB "trans person") OR (TI "trans person")) OR ((AB "trans woman") OR (TI "trans woman")) OR ((AB "trans women") OR (TI "trans women")) OR ((AB "gender identity disorder*") OR (TI "gender identity disorder*")) OR ((AB bicurious) OR (TI bicurious)) OR ((AB intersex*) OR (TI intersex*)) OR ((AB Asexual*) OR (TI Asexual*)) OR ((AB "Two-spirit*") OR (TI "Two- spirit*")) OR ((AB Pansex*) OR (TI Pansex*)) OR ((AB "Gender queer") OR (TI "Gender queer")) OR ((AB Agender*) OR (TI Agender*)) OR ((AB Bigender*) OR (TI Bigender*)) OR ((AB Pangender*) OR (TI Pangender*)) OR ((AB Omnisexual*) OR (TI Omnisexual*)) OR ((AB "Gender variant*") OR (TI "Gender variant*")) OR ((AB "Gender fluid") OR (TI "Gender fluid")) OR ((AB "SOGIE") OR (TI "SOGIE")) OR ((AB "Diverse sexual orientation*") OR (TI "Diverse sexual orientation*")) OR ((AB "Diverse gender*") OR (TI "Diverse gender*"))) AND ((DE "Immigration") OR (DE "Undocumented Immigration") OR

((AB "Emigrants and Immigrants") OR (TI "Emigrants and Immigrants")) OR ((AB "Emigration and Immigration") OR (TI "Emigration and Immigration")) OR (DE "Refugees") OR ((AB Refugee*) OR (TI Refugee*)) OR ((AB "War-Related Injuries") OR (TI "War-Related Injuries")) OR ((AB Newcomer*) OR (TI Newcomer*)) OR ((AB Settler*) OR (TI Settler*)) OR ((AB Noncitizen*) OR (TI Noncitizen*)) OR ((AB "Forced migrant*") OR (TI "Forced migrant*")) OR ((AB Incomer*) OR (TI Incomer*)) OR ((AB Incoming*) OR (TI Incoming*)) OR ((AB Foreign*) OR (TI Foreign*)) OR ((AB Asylum*) OR (TI Asylum*)) OR ((AB Undocumented) OR (TI Undocumented))) AND ((DE "Interpersonal Relationships") OR ((AB "Professional-Patient Relation*") OR (TI "Professional-Patient Relation*")) OR (MM "Patient Centered Care") OR (DE "Health Personnel") OR (DE "Allied Health Personnel") OR (DE "Caregivers") OR (DE "Medical Personnel") OR (DE "Mental Health Personnel") OR ((AB Nurse*) OR (TI Nurse*)) OR ((AB "Mental health professional*") OR (TI "Mental health professional*")) OR ((AB Psychologist*) OR (TI Psychologist*)) OR ((AB Midwi*) OR (TI Midwi*)) OR ((AB Doctor*) OR (TI Doctor*)) OR ((AB Physician*) OR (TI Physician*)) OR ((AB Clinician*) OR (TI Clinician*)) OR ((AB Therapist*) OR (TI Therapist*)) OR ((AB Counselor*) OR (TI Counselor*)) OR ((AB Provider*) OR (TI Provider*)) OR ((AB Professional*) OR (TI Professional*)) OR ((AB "Health Service*") OR (TI "Health Service*"))) AND (((AB "Experience*") OR (TI "Experience*")) OR ((AB "Lived experience*") OR (TI "Lived experience*")) OR ((AB "Perspective*") OR (TI "Perspective*"))) AND (((AB Interview*) OR (TI Interview*)) OR ((AB "Focus group*") OR (TI "Focus group*")) OR ((AB "Discussion*") OR (TI "Discussion*")) OR ((AB "Narrative*") OR (TI "Narrative*")) OR ((AB "Qualitative Stud*") OR (TI "Qualitative Stud*")) OR ((AB "Qualitative research") OR (TI "Qualitative research")) OR ((AB "Qualitative*") OR (TI "Qualitative*")) OR ((AB "Phenomen*") OR (TI "Phenomen*")) OR ((AB "Grounded theor*") OR (TI "Grounded theor*")) OR ((AB "Ethnograph*") OR (TI "Ethnograph*")) OR ((AB "Content analys*") OR (TI "Content analys*")) OR ((AB "Thematic analys*") OR (TI "Thematic analys*")))

Database: SCOPUS
2024-07-20
Hits: 24
Complete search string: TITLE-ABS-KEY((("Sexual and Gender Minorit*") OR (queer*) OR (homosexual*) OR (”sexual minorit*”) OR (”sexual orientation*”) OR (”gender minorit*”) OR (lesbian*) OR (gay) OR (bisexual*) OR (”same sex couple*”) OR (”same sex relation*”) OR (”women loving women”) OR (”men who have sex with men”) OR (”women who have sex with women”) OR (Lesbigay) OR (”Non-Heterosex*”) OR (”Non heterosex*”) OR (GLB) OR (GLBT) OR (GLBTQ) OR (LGB) OR (LGBT) OR (LGBTQ) OR (LGBTQI) OR (LGBTQIA) OR (transgender*) OR (transsexual*) OR (”gender dysphoria”) OR (”gender nonbinary”) OR (”nonbinary gender”) OR (nonbinary) OR (”non binary”) OR (transvesti*) OR (crossgender) OR (”gender change”) OR (”gender transform*”) OR (”gender transition”) OR (genderqueer) OR (”trans female*”) OR (”trans male*”) OR (”trans man”) OR (”trans men”) OR (”trans people”) OR (”trans person”) OR (”trans woman”) OR (”trans women”) OR (”gender identity disorder*”) OR (bicurious) OR (intersex*) OR (Asexual*) OR (”Two- spirit*”) OR (Pansex*) OR (”Gender queer”) OR (Agender*) OR (Bigender*) OR (Pangender*) OR (Omnisexual*) OR (”Gender variant*”) OR (”Gender fluid”) OR (”SOGIE”) OR (”Diverse sexual orientation*”) OR (”Diverse gender*”)) AND (("Emigrants and Immigrants") OR (”Emigration and Immigration”) OR (”Refugees”) OR (Refugee*) OR (”War-Related Injuries”) OR (Newcomer*) OR (Settler*) OR (Noncitizen*) OR (”Forced migrant*”) OR (Incomer*) OR (Incoming*) OR (Foreign*) OR (Asylum*) OR (Undocumented)) AND ((”Interpersonal Relations”) OR (”Professional-Patient

Relations”) OR (“Patient-Centered Care”) OR (”Health Personnel”) OR (Nurse*) OR (”Mental health professional*”) OR (Psychologist*) OR (Midwi*) OR (Doctor*) OR (Physician*) OR (Clinician*) OR (Therapist*) OR (Counselor*) OR (Provider*) OR (Professional*) OR (“Health service*”)) AND ((“Experience*”) OR (“Lived experience*”) OR (“Perspective*”)) AND ((Interview*) OR (”Focus group*”) OR (“Discussion*”) OR (“Narrative*”) OR (”Qualitative research”) OR (“Qualitative*”) OR (“Phenomen*”) OR (“Grounded theor*”) OR (“Ethnograph*”) OR (“Content analys*”) OR (“Thematic analys*”)))
