# Supplementary material for: Sexual and Gender Minority Migrants' Experiences of Health Service Access and Utilisation: A Qualitative Meta‐Synthesis
Source: J Clin Nurs. 2025 Feb 14;34(10):4448–61. doi: 10.1111/jocn.17683 (PMC12409289; doi:10.1111/jocn.17683)
Supplement: Supplementary file 5 — File S5. [file JOCN-34-4448-s002.pdf]

Supplementary File 5. Methodological characteristics of included studies (n=21).

Report (year)	Aim/research question	Recruitment	Sample: Total sample (n)	Sexual orientations (n)	Gender identities[1] (n)	Data collection	Analysis	CASP	JBI
Alessi et al. (2020)	How do LGBTQ refugees from Islamic societies describe and understand their integration experiences	Purposeful	38	Gay: 24<br>Lesbian: 3<br>Bisexual: 3	Trans woman: 5<br>Q/GN/NB: 2<br>Trans man: 1<br>Not presented: 30	Semi-structured interviews	Thematic analysis	7	9
Alessi (2016)	To explore resilience in sexual and gender minority forced migrants.	Purposeful	26	Gay: 20<br>Lesbian: 2	Trans woman: 2<br>Trans man: 2<br>Not presented: 22	Semi-structured interviews	Thematic analysis	8	9
Attia et al. (2022)	Explored the experiences of LGBTQ+ asylum seekers, to answer the research question: What are the pre- and post-migration experiences of LGBTQ+ asylum seekers?	Convenience	8	Not presented: 8	Cisgender woman: 4<br>Cisgender man: 3<br>Transgender (not specified): 1	Semi-structured interviews	Thematic analysis	6	9
Attia et al. (2023)	Address gap in the field's understanding of resilience among LGBTQ+ asylum seekers that might not align with the existing resilience paradigm, to answer the research question: What are the experiences of post-traumatic growth and resilience among LGBTQ+ asylum seekers.	Convenience	7	Not presented: 7	Cisgender woman: 3<br>Cisgender man: 3<br>Transgender (not specified): 1	Semi-structured interviews, Follow-up interviews	Interpretative phenomenological analysis	6	9
Cox et al. (2022)	To explore the experiences of discrimination and its impacts on well-being among racialised LGBTQ+ newcomers living in a small urban area and representing a variety of intersecting identities.	Convenience, Snowball	10	Bisexual: 4<br>Gay: 2<br>Lesbian: 2<br>Queer: 1<br>Asexual: 1	Cisgender woman: 5<br>Cisgender man: 5	Semi-structured interviews, Member checking session	Thematic analysis	9	10
Haghiri-Vijeh (2022)	What have been the experiences of LGBTQIA+ migrants in their interactions with nurses and other healthcare professionals?	Convenience	16	Gay: 8<br>Lesbian: 2<br>Queer: 2<br>Bisexual: 1<br>Pansexual: 1<br>Not presented: 2	Cisgender man: 10<br>Q/GNC/NB: 5<br>Trans woman: 1	Semi-structured interviews	Hermeneutic analysis	7	9
Kahn et al. (2018)	To understand the facilitators of and barriers to mental health care for LGBT forced migrants. Research questions: (a) How do gateway providers (legal providers, advocates, resettlement workers, and private sponsors), mental health providers, and LGBT forced migrants describe the challenges of connecting with mental health care, and (b) To what extent do other avenues of support supplement individual mental health treatment for LGBT forced migrants?	Purposeful	7	Gay: 4<br>Lesbian: 2<br>Not presented: 1	Transgender (not specified): 1<br>Not presented: 6	Semi-structured interviews	Thematic analysis	8	9

Kahn (2014)	To elucidate relationships between Muslim asylum seekers and Islamic faith. Research questions: Whether, and how, was faith connected to expectations for gender role conformity in their societies of origin? What were their experiences of reconciling faith with their gender role non-conformity? How did they subsequently experience relationships with God post asylum? How did social workers or other mental health and social service providers assist or facilitate explorations of religious faith and affiliation?	Purposeful	7	Gay: 7	Cisgender man: 7	Semi-structured interviews, 2 interviews per participant	Constant comparative analysis/ grounded theory	7	7
Koskan & Fernandez-Pineda (2018)	To explore understanding of primary and secondary prevention of anal cancer among human immunodeficiency virus-infected foreign-born Latino gay and bisexual men.	Convenience	33	Not presented (only gay or bisexual): 33	Not presented: 33	Semi-structured interviews	Content analysis	8	9
Lee et al. (2023)	To elicit perspectives on how transgender Latina immigrants' multiple identities, social processes, and the current socio-political climate, shape health needs and experiences of health access.	Convenience	10	Heterosexual: 5<br>Gay: 3<br>Other: 1<br>Pansexual: 1	Trans woman: 10	Semi-structured interviews	Thematic analysis	7	7
Logie et al. (2016)	To explore experiences of social support group participation among LGBT African and Caribbean newcomers and refugees in an urban city.	Convenience, Snowball	29	Bisexual: 12<br>Gay: 9<br>Lesbian: 5<br>Heterosexual: 1<br>Other: 1<br>Not presented: 1	Cisgender man: 15<br>Cisgender woman: 11<br>Transgender (not specified): 3	Focus group discussions	Thematic analysis	8	8
Brooks et al. (2024)	To identify distinct barriers and facilitators immigrant Latino men who have sex with men's experience accessing sexual health services given their complex intersectional identities of being an immigrant, Latino, and a sexual minority man.	Purposeful	25	Gay: 22<br>Bisexual: 2<br>Queer: 1	Cisgender man: 25	Semi-structured interviews	Thematic analysis	8	8
Carlsson et al. (2024)	To explore experiences of social and health professional support among sexual minority forced migrant men.	Convenience, Purposeful, Snowball	15	Gay: 14<br>Bisexual: 1	Cisgender man: 15	Semi-structured interviews	Thematic analysis	9	10
Fuks et al. (2018)	To generate in-depth understanding of the migration and acculturation process among LGBT immigrants. Research question: "How do LGBT immigrants perceive their process of acculturation.	Convenience, Purposeful, Snowball	20	Gay: 10<br>Bisexual: 4<br>Lesbian: 2<br>Heterosexual: 2<br>Queer: 2	Cisgender man: 12<br>Cisgender woman: 5<br>Transgender (not specified): 3	Semi-structured interviews	Constant comparative analysis/ grounded theory	7	8

Van Landeghem et al. (2023)	To explore underlying determinants to pre-exposure prophylaxis (PrEP) acceptance and uptake and to investigate the factors and cross-cutting mechanisms that either hinder or facilitate PrEP uptake and use among migrant men and transwomen who have sex with men.	Via clinical workers (not specified further)	23	Have sex with men: 23	Cisgender man: 22<br>Trans woman: 1	Semi-structured interviews	Constant comparative analysis/ grounded theory	9	10
Navaza et al. (2016)	Examined HIV testing experiences and perceptions amongst Latin-American migrant men who have sex with men and transgender females.	Purposeful	38	Have sex with men: 38	Cisgender man: 25<br>Trans woman: 13	Semi-structured interviews, Focus group discussions	Thematic analysis	8	8
Mulé (2022)	To examine the mental health issue and needs of LGBTQ asylum seekers and refugees from a critical psychology perspective. The aim was to critically understand how LGBTQ asylum seekers and refugees are impacted by their experience. Research questions: Did they have adequate access to information and resources? How did they manage socio-cultural shifts in understanding the concept of identities? How did they navigate the refugee claims process? How did they manage trauma? How has their experience impacted their mental health?	Convenience	92	Gay: 38<br>Bisexual: 24<br>Lesbian: 18<br>Heterosexual: 2<br>Queer: 2<br>Pansexual: 1<br>Not presented. 7	Cisgender man: 52<br>Cisgender woman: 33<br>Transgender (not specified): 7<br>Two-spirit: 2<br>Not presented: 2	Focus group discussions	Participatory action research	7	7
Munro et al. (2013)	To explore the nature of various forms of oppression experienced by LGBT newcomers and offer recommendations for transforming services to better serve the complex needs of this marginalized population.	Convenience	40	Gay: 16<br>Queer: 10<br>Lesbian: 6<br>Bisexual: 4<br>Pansexual: 3<br>Not presented: 1	Cisgender man: 27<br>Cisgender woman: 10<br>Trans woman: 1<br>Q/GNC/NB: 2	Focus group discussions	Thematic analysis	6	7
Oren & Gorshkov (2021)	To explore and interpret lived experience of the Russian-speaking LGBT+ immigrants.	Purposeful, Snowball	9	Not presented. 9	Not presented: 9	Semi-structured interviews	Interpretative phenomenological analysis	9	10
Philpot et al. (2022)	To describe gay and bisexual migrants' reactions to their diagnosis and their immediate and long-term expectations for, and concerns about, living with HIV.	Not specified	24	Gay: 21<br>Bisexual: 3	Cisgender man: 24	Semi-structured interviews	Thematic analysis	7	10
Rhodes et al. (2015)	To explore needs, assets, and priorities of Latina transgender women.	Convenience	9	Not presented: 9	Trans woman: 9	Photovoice	Constant comparative analysis/ grounded theory	8	7

[1]Some participants may have reported more than one sexual orientation and/or gender identity. Q/GNC/NB: Queer, gender non-conforming, and/or non-binary gender identity.
