# Supplementary material for: Sexual and Gender Minority Migrants' Experiences of Health Service Access and Utilisation: A Qualitative Meta‐Synthesis
Source: J Clin Nurs. 2025 Feb 14;34(10):4448–61. doi: 10.1111/jocn.17683 (PMC12409289; doi:10.1111/jocn.17683)
Supplement: Supplementary file 6 — File S6. [file JOCN-34-4448-s003.pdf]

**Supplementary File 6.** Expanded presentation of results of meta-synthesis.
The meta-synthesis resulted in two overarching themes: *Left out of needed support: encountering barriers to accessing health services* and *Between healing and harm: the dual faces of interactions in health services*. Results are presented with frequency effect sizes (FES).

4.3.1 Left out of needed support: encountering barriers to accessing health services
The theme barriers hindering access to healthcare included *Excluded from care based on external barriers to health services* and *Struggling in silence while dealing with internal barriers to health services.* Barriers to healthcare impacted the mental and physical health of migrants (FES 14%) (Carlsson et al., 2024; Mulé, 2022; Rhodes et al., 2015).

4.3.1.1 Excluded from care based on external barriers to health services
Various external structural barriers related to intersectional identities hindered access to healthcare (FES 81 %) (Attia et al., 2022; Attia et al., 2023; Brooks et al., 2024; Carlsson et al., 2024; Cox et al., 2022; Fuks et al., 2018; Haghiri-Vijeh, 2022; Kahn, 2014; Kahn et al., 2018; Koskan & Fernandez-Pineda, 2018; Lee et al., 2023; Logie et al., 2016; Mulé, 2022; Munro et al., 2013; Navaza et al., 2016; Oren & Gorshkov, 2021; Rhodes et al., 2015; Van Landeghem et al., 2023). **Stigma and culture** hindered access to healthcare (FES 38%) (Attia et al., 2022; Attia et al., 2023; Brooks et al., 2024; Fuks et al., 2018; Kahn et al., 2018; Koskan & Fernandez-Pineda, 2018; Lee et al., 2023; Van Landeghem et al., 2023), including *stigma around mental health treatment* (FES 24%) (Attia et al., 2022; Attia et al., 2023; Fuks et al., 2018; Koskan & Fernandez-Pineda, 2018; Lee et al., 2023), *HIV as a gay man* (FES 14%) (Brooks et al., 2024; Koskan & Fernandez-Pineda, 2018; Van Landeghem et al., 2023), and *cultural norms around masculinity* (FES 10%) (Kahn et al., 2018; Koskan & Fernandez-Pineda, 2018). **Financial constraints** hindered access to healthcare (FES 48%) (Attia et al., 2023; Brooks et al., 2024; Carlsson et al., 2024; Haghiri-Vijeh, 2022; Koskan & Fernandez-Pineda, 2018; Lee et al., 2023; Mulé, 2022; Oren & Gorshkov, 2021; Rhodes et al., 2015; Van Landeghem et al., 2023), associated with *high healthcare costs* (FES 33%) (Brooks et al., 2024; Carlsson et al., 2024; Haghiri-Vijeh, 2022; Lee et al., 2023; Mulé, 2022; Rhodes et al., 2015; Van Landeghem et al., 2023), *lack of health insurance* (FES 29%) (Attia et al., 2023; Brooks et al., 2024; Koskan & Fernandez-Pineda, 2018; Lee et al., 2023; Oren & Gorshkov, 2021; Rhodes et al., 2015), and *ineligibility for financial assistance* (FES 5%) (Brooks et al., 2024). The financial constraints led to risky health behaviours and unregulated self-medication (FES 10%) (Attia et al., 2023; Rhodes et al., 2015). **Lack of information dissemination** further hindered access to healthcare (FES 48%) (Brooks et al., 2024; Carlsson et al., 2024; Fuks et al., 2018; Kahn et al., 2018; Koskan & Fernandez-Pineda, 2018; Lee et al., 2023; Logie et al., 2016; Navaza et al., 2016; Rhodes et al., 2015; Van Landeghem et al., 2023), including *how to access a complex healthcare system* (FES 29%) (Brooks et al., 2024; Carlsson et al., 2024; Kahn et al., 2018; Lee et al., 2023; Logie et al., 2016; Van Landeghem et al., 2023), *prevention of infections and anal cancer* (FES 24%) (Brooks et al., 2024; Fuks et al., 2018; Koskan & Fernandez-Pineda, 2018; Navaza et al., 2016; Van Landeghem et al., 2023), and *rights to healthcare when undocumented* (FES 5%) (Carlsson et al., 2024). Migrants expressed a need for health information tailored to their trans identity and language proficiency (FES14%) (Brooks et al., 2024; Lee et al., 2023; Rhodes et al., 2015). **Language barriers** hindered access to healthcare, including having difficulties booking appointments for healthcare visits (FES 19%) (Carlsson et al., 2024; Lee et al., 2023; Mulé, 2022; Van Landeghem et al., 2023). Migrants expressed a **lack of available health services** (FES 38%) (Brooks et al., 2024; Carlsson et al., 2024; Fuks et al., 2018; Kahn et al., 2018; Lee et al., 2023; Navaza et al., 2016; Oren & Gorshkov, 2021; Van Landeghem et al., 2023), specifically

a lack of *mental health services* (FES 14%) (Kahn et al., 2018; Lee et al., 2023; Oren & Gorshkov, 2021), *LGBTQ+ specialized services* (FES 14%) (Brooks et al., 2024; Fuks et al., 2018; Lee et al., 2023), and *language appropriate services* (FES 14%) (Brooks et al., 2024; Lee et al., 2023; Oren & Gorshkov, 2021).They also experienced long waiting for health service appointments (FES 14%) (Carlsson et al., 2024; Navaza et al., 2016; Van Landeghem et al., 2023). **Living undocumented** was a barrier for access to many healthcare services (FES 19%) (Carlsson et al., 2024; Mulé, 2022; Munro et al., 2013; Van Landeghem et al., 2023).

*Participants described the multitude of barriers they face in accessing care. Emiliana noted that "the very high cost" of mental health services is a factor that limits members of their community from receiving them. For Adriana, cost and lack of insurance were also major challenges to accessing any type of mental health support.* (Lee et al., 2023)

4.3.1.2 Struggling in silence while dealing with internal barriers to health services

Migrants expressed internal barriers hindering access to healthcare (FES 57%) (Alessi et al., 2020; Brooks et al., 2024; Carlsson et al., 2024; Cox et al., 2022; Fuks et al., 2018; Haghiri-Vijeh, 2022; Kahn, 2014; Kahn et al., 2018; Koskan & Fernandez-Pineda, 2018; Lee et al., 2023; Navaza et al., 2016; Philpot et al., 2022; Van Landeghem et al., 2023). **Fears** hindered migrants from accessing healthcare (FES 38%) (Alessi et al., 2020; Brooks et al., 2024; Carlsson et al., 2024; Cox et al., 2022; Fuks et al., 2018; Koskan & Fernandez-Pineda, 2018; Philpot et al., 2022; Van Landeghem et al., 2023), including fears related to *being reported to authorities by health professionals* (FES 19%) (Brooks et al., 2024; Carlsson et al., 2024; Koskan & Fernandez-Pineda, 2018; Van Landeghem et al., 2023), *being judged by health professionals and others in the healthcare setting* (FES 19%) (Cox et al., 2022; Fuks et al., 2018; Koskan & Fernandez-Pineda, 2018; Van Landeghem et al., 2023), *screening discomfort and potential test results* (FES 5%) (Koskan & Fernandez-Pineda, 2018), and *fear of being denied gender affirming care if deported* (Alessi et al., 2020). *Internalized stigma and prior experiences in country of origin* contributed to fears seeking care in the host country (FES 14%) (Brooks et al., 2024; Fuks et al., 2018; Philpot et al., 2022). *Intersectional layers further contributed to fears* of engaging with health services (FES 5%) (Koskan & Fernandez-Pineda, 2018) and migrants were more inclined to access health services when *health professionals matched one or more of their identities* (FES 5%) (Brooks et al., 2024). Having **prior experiences of discrimination in healthcare** led to migrants avoiding further contact (FES 24%) (Brooks et al., 2024; Carlsson et al., 2024; Haghiri-Vijeh, 2022; Lee et al., 2023; Van Landeghem et al., 2023). **Shame** (FES 14%) (Carlsson et al., 2024; Kahn et al., 2018; Lee et al., 2023) and **embarrassment** (FES 14%) (Brooks et al., 2024; Koskan & Fernandez-Pineda, 2018; Navaza et al., 2016) further hindered access to healthcare.

*[Immigrant Latino men who have sex with men] were hesitant to access [HIV] services because they feared that their personal identifiable information would be reported to or shared with immigration authorities, the State government, or employers. [...] also feared that accessing these services would negatively impact the immigration process and lead to their visa being revoked.* (Brooks et al., 2024)

4.3.2 Between healing and harm: the dual faces of interactions in health services

The theme included the sub-themes *Care becomes a burden when facing non-affirming behaviours and discrimination* (FES 24%) (Brooks et al., 2024; Carlsson et al., 2024; Haghiri-Vijeh, 2022; Lee et al., 2023; Navaza et al., 2016) and *The power of acceptance and*

*affirmation when meeting health professionals* (FES 62%) (Alessi, 2016; Attia et al., 2023; Brooks et al., 2024; Carlsson et al., 2024; Cox et al., 2022; Haghiri-Vijeh, 2022; Kahn, 2014; Kahn et al., 2018; Lee et al., 2023; Navaza et al., 2016; Philpot et al., 2022; Rhodes et al., 2015; Van Landeghem et al., 2023).

*4.3.2.1 Care becomes a burden when facing non-affirming behaviours and discrimination*
Migrants encountered non-affirming behaviours and discrimination based on both their SGM identity as well as migration and ethnicity/race. Covert and overt acts of discrimination were outlined (FES 24%) (Brooks et al., 2024; Carlsson et al., 2024; Haghiri-Vijeh, 2022; Lee et al., 2023; Navaza et al., 2016). A **power imbalance between health professionals and migrants** were reported, with migrants recounting being asked *oppressive irrelevant questions* and *instances of sexual harassment* (FES 5%) (Haghiri-Vijeh, 2022). Non-affirming and discriminating encounters with health professionals had a **negative impact on the health and wellbeing** of migrants (FES 14%) (Carlsson et al., 2024; Haghiri-Vijeh, 2022; Lee et al., 2023). Non-affirming behaviours, discrimination, transphobic attitudes, and lack of sexual education had a tra*umatizing impact and made migrants relive previous traumas* (FES 5%) (Haghiri-Vijeh, 2022). These instances compounded feelings of *shame, hesitancy, inferiority, frustration, and feeling neglected* (FES 14%) (Carlsson et al., 2024; Haghiri-Vijeh, 2022; Lee et al., 2023).

*Participants felt that some nurses and other health professionals spoke from a position of power and privilege and asked invasive and unnecessary questions*. (Haghiri-Vijeh, 2022)

Gender minority migrants encountered **trans-specific non-affirming behaviours and discrimination** (FES 10%) (Haghiri-Vijeh, 2022; Lee et al., 2023), including *misgendering, refusal to use correct pronouns, using names assigned at birth* (FES 5%) (Haghiri-Vijeh, 2022), *degrading comments*, and *intrusive questions* (FES 10%) (Haghiri-Vijeh, 2022; Lee et al., 2023). Transgender participants expressed *unaddressed health needs* based on not being listened to, understood, and accepted by health professionals (FES 10%) (Haghiri-Vijeh, 2022; Lee et al., 2023). Migrants also experienced **racist behaviours from health professionals**, which resulted in them feeling *mistreated* and *avoiding seeking further healthcare* (FES 14%) (Carlsson et al., 2024; Haghiri-Vijeh, 2022; Lee et al., 2023).

*Participants reported experiences of being dead named and misgendered by a wide variety of nurses and other healthcare professionals [...] these brought back memories of trauma. For example, even after correcting the care provider, Ali, a trans migrant participant, was outed in the waiting room.* (Haghiri-Vijeh, 2022)

**Less overt forms of discrimination** were encountered (FES 24%) (Brooks et al., 2024; Carlsson et al., 2024; Haghiri-Vijeh, 2022; Lee et al., 2023; Navaza et al., 2016), including *not feeling welcomed and accepted* (FES 19%) (Brooks et al., 2024; Carlsson et al., 2024; Lee et al., 2023; Navaza et al., 2016), g*etting looks from health professionals* (FES 5%) (Navaza et al., 2016), and *feeling mistreated based on language proficiency* (FES 5%) (Haghiri-Vijeh, 2022). Moreover, migrants faced **inattentiveness from health professionals**, experiencing them as *rushed and dismissive* (FES 10%) (Carlsson et al., 2024; Haghiri-Vijeh, 2022). They also expressed a **lack of knowledge among health professionals** regarding the health needs of SGM individuals (FES 5%) (Carlsson et al., 2024).

*Some health professionals had been in a hurry during their appointments and were inattentive*

*to their needs. Additionally, some health professionals had not addressed topics related to sexual health and were experienced as having insufficient knowledge about the health needs of sexual minorities. (Carlsson et al., 2024)*

5.3.2.2 *The power of acceptance and affirmation when meeting health professionals*
**Migrants appreciated the support received from health services** (FES 24%) (Attia et al., 2023; Brooks et al., 2024; Haghiri-Vijeh, 2022; Navaza et al., 2016; Rhodes et al., 2015), such as help *navigating the system* (FES 24%) (Attia et al., 2023; Brooks et al., 2024; Haghiri-Vijeh, 2022; Navaza et al., 2016; Rhodes et al., 2015) and being *offered follow-up communication after health visits* (FES 5%) (Haghiri-Vijeh, 2022). **Engaging with health services benefited health in many ways** (FES 24%) (Alessi, 2016; Attia et al., 2023; Haghiri-Vijeh, 2022; Kahn, 2014; Lee et al., 2023), including *relief of post-traumatic stress symptoms* (FES 10%) (Alessi, 2016; Kahn, 2014), *anxiety* (FES 10%) (Alessi, 2016; Haghiri-Vijeh, 2022), *sleeping difficulties* (FES 5%) (Alessi, 2016), *pain* (FES 5%) (Haghiri-Vijeh, 2022), and *fears* (FES 5%) (Haghiri-Vijeh, 2022). Mental health support improved *resilience and hope* (FES 19%) (Alessi, 2016; Attia et al., 2023; Haghiri-Vijeh, 2022; Kahn, 2014), *social stability* (FES 5%) (Kahn, 2014), *wellbeing* (FES 5%) (Lee et al., 2023), *relaxation* (FES 10%) (Haghiri-Vijeh, 2022; Philpot et al., 2022), and helped *explore tensions between gender identity and faith* (FES 5%) (Kahn, 2014). However, *not all migrants felt that therapy helped improve their resilience* (FES 5%) (Alessi, 2016).

*All ultimately found the process of interacting with a mental health professional to be helpful in restoring hope and mitigating distress associated with past persecution and the psychological and social impacts of flight, asylum and resettlement.* (Kahn, 2014)

**The characteristics of health professionals were recognized as key to establishing client-professional connections** (FES 33%) (Alessi, 2016; Brooks et al., 2024; Carlsson et al., 2024; Haghiri-Vijeh, 2022; Kahn et al., 2018; Navaza et al., 2016; Van Landeghem et al., 2023). Characteristics that could have a negative effect on connection included the *personality and behavior of professionals* (FES 24%) (Alessi, 2016; Carlsson et al., 2024; Kahn et al., 2018; Navaza et al., 2016; Van Landeghem et al., 2023), *when professionals did not share the migrants' SGM identity* (FES 14%) (Brooks et al., 2024; Haghiri-Vijeh, 2022; Kahn et al., 2018), *cultural differences* (FES 5%) (Alessi, 2016), and *when professionals originated from the same country* (FES 5%) (Haghiri-Vijeh, 2022). **Competence development in intersectional identities and support for transgender individuals was emphasized** (FES 10%) (Cox et al., 2022; Rhodes et al., 2015), along with the **importance of respectful** (FES 24%) (Brooks et al., 2024; Carlsson et al., 2024; Haghiri-Vijeh, 2022; Rhodes et al., 2015; Van Landeghem et al., 2023) and **affirming health services** (FES 10%) (Carlsson et al., 2024; Van Landeghem et al., 2023). Participants appreciated **compassionate**, **friendly**, and **encouraging** health professionals (FES 29%) (Alessi, 2016; Carlsson et al., 2024; Haghiri-Vijeh, 2022; Navaza et al., 2016; Philpot et al., 2022; Rhodes et al., 2015) who **accepted them, validated their experiences** (FES 14%) (Brooks et al., 2024; Haghiri-Vijeh, 2022; Philpot et al., 2022), **understood them**, and **affirmed them** (FES 10%) (Carlsson et al., 2024; Van Landeghem et al., 2023). They **needed a safe** (FES 5%) (Brooks et al., 2024) and **welcoming space** (FES 10%) (Carlsson et al., 2024; Rhodes et al., 2015) where they could discuss their sexual health in a *non-judgmental setting*. **Openness** was crucial (FES 24%) (Brooks et al., 2024; Carlsson et al., 2024; Haghiri-Vijeh, 2022; Kahn, 2014; Van Landeghem et al., 2023), with *the need for open-minded professionals* (FES 10%) (Carlsson et al., 2024; Haghiri-Vijeh, 2022) *willing to learn from them* (FES 5%) (Carlsson et al., 2024), *respect*

*their faith* (FES 5%) (Kahn, 2014), and who *enable free discussion of questions* (FES 10%) (Brooks et al., 2024; Van Landeghem et al., 2023).

*The participants felt heard and accepted when nurses and other healthcare professionals listened attentively, respected them, were aware of their past traumatic experiences, and provided compassionate, caring, kind, and nonjudgmental care.* (Haghiri-Vijeh, 2022)

Migrants were **concerned about disclosure and confidentiality** when interacting with health services (FES 29%) (Alessi, 2016; Brooks et al., 2024; Carlsson et al., 2024; Haghiri-Vijeh, 2022; Navaza et al., 2016; Philpot et al., 2022), *worrying about consequences if sensitive data was leaked from health journals* (FES 10%) (Navaza et al., 2016; Philpot et al., 2022). *Waiting rooms and receptions were spaces where their identity could be exposed*, requiring sensitivity from professionals (FES 5%) (Philpot et al., 2022). *Heteronormative assumptions among professionals* (FES 5%) (Haghiri-Vijeh, 2022) and *cultural stigma* (FES 10%) (Alessi, 2016; Haghiri-Vijeh, 2022) hindered disclosure. Migrants *worried how professionals would react if they disclosed their identity* (FES 10%) (Carlsson et al., 2024; Haghiri-Vijeh, 2022). To avoid visibility and feel comfortable, *some decided to conceal their SGM identity* (FES 5%) (Haghiri-Vijeh, 2022) and *preferred specialized health services* (FES 5%) (Brooks et al., 2024).

*There were concerns that being seen in the waiting room might arouse assumptions from other patients about their HIV status, thus implicitly contravening confidentiality. [...] His concerns about waiting room confidentiality were drawn from his experiences in his country of origin, where presence in waiting rooms in sexual health clinics could attract gossip from others in the community.* (Philpot et al., 2022)

**Language barriers** made it difficult to express health concerns (FES 14%) (Brooks et al., 2024; Carlsson et al., 2024; Van Landeghem et al., 2023), with migrants *stating a need for adequate interpreter services* (FES 10%) (Brooks et al., 2024; Carlsson et al., 2024). However, various **reservations and doubts were articulated regarding the utilization of interpreters** (FES 19%) (Brooks et al., 2024; Carlsson et al., 2024; Haghiri-Vijeh, 2022; Lee et al., 2023). This included feel*ing uncomfortable and embarrassed when several people were in the room* (FES 19%) (Brooks et al., 2024; Carlsson et al., 2024; Haghiri-Vijeh, 2022; Lee et al., 2023), *fearing how the interpreter would react* (FES 5%) (Carlsson et al., 2024), and *the potential risk of sensitive information being spread through the interpreter* (FES 5%) (Carlsson et al., 2024). Consequently, some *preferred telephonic interpretation* to remain anonymous (FES 5%) (Carlsson et al., 2024).

*Participants felt that the translation services provided were inadequate or created situations where they were either uncomfortable or embarrassed discussing their sexual behaviors with multiple individuals.* (Brooks et al., 2024)

Alessi, E. J. (2016). Resilience in sexual and gender minority forced migrants: a qualitative exploration. *Traumatology*, *22*(3), 203-213. https://doi.org/10.1037/trm0000077

Alessi, E. J., Kahn, S., Greenfield, B., Woolner, L., & Manning, D. (2020). A Qualitative Exploration of the Integration Experiences of LGBTQ Refugees Who Fled from the Middle East, North Africa, and Central and South Asia to Austria and the Netherlands. *Sexuality Research and Social Policy*, *17*(1), 13-26. https://doi.org/10.1007/s13178-018-0364-7

Attia, M., Das, B., Tang, S., Li, H., & Qiu, Y. (2022). Pre- and Post-Migration Experiences of LGBTQ+ Asylum-Seeking Individuals: A Phenomenological Investigation. *Journal of LGBTQ Issues in Counseling*, *16*(3), 207-225. https://doi.org/10.1080/26924951.2022.2043216

Attia, M., Das, B., Tang, S., Qiu, Y., Li, H., & Nguyen, C. (2023). Post-Traumatic Growth and Resilience of LGBTQ+ Asylum Seekers in the United States. *The Counseling Psychologist*, *51*(7), 1005-1036. https://doi.org/10.1177/00110000231186112

Brooks, R. A., Nieto, O., Rosenberg-Carlson, E., Morales, K., Üsküp, D. K., Santillan, M., & Inzunza, Z. (2024). Barriers and Facilitators to Accessing PrEP and Other Sexual Health Services Among Immigrant Latino Men Who Have Sex with Men in Los Angeles County. *Arch Sex Behav*. https://doi.org/10.1007/s10508-024-02928-z

Carlsson, T., Isaac, R., Ainembabazi, R., Eldebo, A., Yasin, S., & Gottvall, M. (2024). Desiring support on a winding road with challenging intersections: Social and professional support for sexual minority forced migrant men. *J Adv Nurs*. https://doi.org/10.1111/jan.16256

Cox, E., Warren, T., Khan, M., Wilson, C., Cameron, R., Davis, C., Coleman, T., Steffler, J., Coulombe, S., & Woodford, M. (2022). Experiences of discrimination and its impacts on well-being among racialised LGBTQ+ newcomers living in Waterloo region, Ontario, Canada [Article]. *Health and Social Care in the Community*, *30*(5), e2980-e2988. https://doi.org/10.1111/hsc.13743

Fuks, N., Smith, N. G., Peláez, S., De Stefano, J., & Brown, T. L. (2018). Acculturation Experiences Among Lesbian, Gay, Bisexual, and Transgender Immigrants in Canada. *The Counseling Psychologist*, *46*(3), 296-332. https://doi.org/10.1177/0011000018768538

Haghiri-Vijeh, R. (2022). Experiences of LGBTQIA+ migrants with nurses and other healthcare professionals in Canada. *Nurs Forum*, *57*(6), 1184-1192. https://doi.org/10.1111/nuf.12819

Kahn, S. (2014). Experiences of Faith for Gender Role Non-Conforming Muslims in Resettlement: Preliminary Considerations for Social Work Practitioners. *The British Journal of Social Work*, *45*(7), 2038-2055. https://doi.org/10.1093/bjsw/bcu060

Kahn, S., Alessi, E. J., Kim, H., Woolner, L., & Olivieri, C. J. (2018). Facilitating Mental Health Support for LGBT Forced Migrants: A Qualitative Inquiry [Article]. *Journal of Counseling and Development*, *96*(3), 316-326. https://doi.org/10.1002/jcad.12205

Koskan, A. M., & Fernandez-Pineda, M. (2018). Anal Cancer Prevention Perspectives Among Foreign-Born Latino HIV-Infected Gay and Bisexual Men [Article]. *Cancer Control*, *25*(1). https://doi.org/10.1177/1073274818780368

Lee, J. J., Leyva Vera, C. A., Ramirez, J., Munguia, L., Herrera, J. A., Basualdo, G., Small, L., & Robles, G. (2023). 'They already hate us for being immigrants and now for being trans-we have double the fight': a qualitative study of barriers to health access among transgender Latinx immigrants in the United States. *J Gay Lesbian Ment Health*, *27*(3), 319-339. https://doi.org/10.1080/19359705.2022.2067279

Logie, C. H., Lacombe-Duncan, A., Lee-Foon, N., Ryan, S., & Ramsay, H. (2016). "It's for us -newcomers, LGBTQ persons, and HIV-positive persons. You feel free to be": a qualitative study exploring social support group participation among African and Caribbean lesbian, gay, bisexual and transgender newcomers and refugees in Toronto, Canada. *BMC Int Health Hum Rights*, *16*(1), 18. https://doi.org/10.1186/s12914-016-0092-0

Mulé, N. J. (2022). Mental health issues and needs of LGBTQ+ asylum seekers, refugee claimants and refugees in Toronto, Canada. *Psychology & Sexuality*, *13*(5), 1168-1178. https://doi.org/10.1080/19419899.2021.1913443

Munro, L., Travers, R., St. John, A., Klein, K., Hunter, H., Brennan, D., & Brett, C. (2013). A bed of roses?: exploring the experiences of LGBT newcomer youth who migrate to Toronto. *Ethnicity and Inequalities in Health and Social Care*, *6*(4), 137-150. https://doi.org/10.1108/EIHSC-09-2013-0018

Navaza, B., Abarca, B., Bisoffi, F., Pool, R., & Roura, M. (2016). Provider-Initiated HIV Testing for Migrants in Spain: A Qualitative Study with Health Care Workers and Foreign-Born Sexual Minorities. *PLoS One*, *11*(2), e0150223. https://doi.org/10.1371/journal.pone.0150223

Oren, T., & Gorshkov, A. (2021). Lived Experiences of Recent Russian-Speaking LGBT+ Immigrants in the United States: An Interpretive Phenomenological Analysis. *Journal of LGBTQ Issues in Counseling*, *15*(3), 290-309. https://doi.org/10.1080/15538605.2021.1914278

Philpot, S. P., Aung, E., Templeton, D. J., Stackpool, G., Varma, R., Power, C., Robinson, S., Stratigos, A., Mao, L., Grulich, A. E., & Bavinton, B. R. (2022). Experiences of recently HIV-diagnosed gay and bisexual migrants in Australia: Implications for sexual health programmes and health promotion. *Health Soc Care Community*, *30*(6), e5801-e5810. https://doi.org/10.1111/hsc.14011

Rhodes, S. D., Alonzo, J., Mann, L., M. Simán, F., Garcia, M., Abraham, C., & Sun, C. J. (2015). Using Photovoice, Latina Transgender Women Identify Priorities in a New Immigrant-Destination State. *International Journal of Transgenderism*, *16*(2), 80-96. https://doi.org/10.1080/15532739.2015.1075928

Van Landeghem, E., Dielen, S., Semaan, A., Rotsaert, A., Vanhamel, J., Masquillier, C., Wouters, E., Wouters, K., Vuylsteke, B., Reyniers, T., & Nöstlinger, C. (2023). Insights into barriers and facilitators in PrEP uptake and use among migrant men and transwomen who have sex with men in Belgium. *BMC Public Health*, *23*(1), 712. https://doi.org/10.1186/s12889-023-15540-y
